# Global mapping of disaggregated international trade-linked transportation CO_2_ emissions

**DOI:** 10.1126/sciadv.adz1670

**Published:** 2026-04-03

**Authors:** Zhenyu Luo, Yuhan Soo, Zhaofeng Lv, Wei Cong, Xiaotong Wang, Shen Qu, Tingkun He, Wen Yi, Chun Deng, Yongshun Xie, Huan Liu, Kebin He

**Affiliations:** ^1^ State Key Laboratory of Regional Environment and Sustainability, School of Environment, Tsinghua University, Beijing 100084, China.; ^2^ Center for Energy and Environmental Policy Research, Beijing Institute of Technology, Beijing 100081, China.; ^3^ School of Management and Economics, Beijing Institute of Technology, Beijing 100081, China.

## Abstract

A comprehensive understanding of international trade-linked transportation CO_2_ emissions is essential for achieving net-zero emission goals. However, the current simplified representation of transportation patterns obscures the heterogeneity of these CO_2_ emissions in international trade and limits the development of targeted decarbonization policies. This study developed an integrated and highly detailed model that incorporated commodity-scale modal shares and shipping carbon intensities for each trade pair, assisted by machine learning and observed voyage signals, respectively. The results indicate that transportation modal shares vary considerably across different scales. In 2021, trade-linked transportation contributed 971 million tonnes of CO_2_ emissions globally. When attributing CO_2_ emissions to countries and commodities, the simplification of modal share can lead to substantial biases through carbon-intensity weighting. By shifting the focus from end-of-pipe emissions to upstream demand, this study identified a decarbonization potential of 41.6% through optimizing transportation distances. The findings offer valuable insights for designing targeted mitigation policies for international freight transportation.

## Teaser

Multiscale trade-linked transportation CO_2_ emissions reveal carbon responsibility allocation biases and decarbonization potential.

## INTRODUCTION

International freight transportation stands as the cornerstone of the global economy, connecting markets and supply chains across continents (*1*, *2*). Among the various transportation modes, shipping, aviation, roads, and railways facilitate most cross-border shipments, and the fossil fuels used in these activities produce substantial CO_2_ emissions. In 2022, international transportation has recovered from the impact of COVID-19 and transported over 100 billion tonne-km of freight worldwide (*3*). This rebound occurred faster than expected, and projections indicate that international trade will continue to grow stably (*4*).

As a cornerstone of the Paris Agreement, the Nationally Determined Contributions do not include carbon emissions from international freight transportation (*5*). This omission represents a notable gap in efforts to mitigate international transportation emissions (*6*). Emission reductions in international transportation are currently driven through global voluntary agreements. For example, the International Civil Aviation Organization (ICAO) and the International Maritime Organization (IMO) have assumed responsibility for reducing greenhouse gas (GHG) emissions in the aviation and shipping sectors. In 2022, ICAO member states agreed to a net-zero CO_2_ emissions goal for international aviation by 2050 (*7*). In 2023, IMO adopted a revised GHG strategy to reach net-zero GHG emissions by or close to 2050, taking into account different national circumstances (*8*). Their strategies encompass measures such as enhancing energy efficiency, optimizing operations, and promoting alternative fuels (*9*–*13*). However, these climate targets involve only limited commitments to new technologies, and since both organizations lack enforcement authority, the implementation of concrete emission reduction actions relies largely on voluntary actions by member states (*14*, *15*).

The transportation sector is one of the most challenging sectors for carbon reduction efforts globally due to the cross-border issues and ambiguous responsibility attribution (*16*). Therefore, a comprehensive understanding of the transportation CO_2_ emissions produced by global international trade is crucial to achieving net-zero emission goals. However, the CO_2_ emissions embedded in international trade transportation exhibit considerable complexity due to varying transportation modes and their differing carbon intensities. The former shapes intricate long-distance transportation patterns, while the latter amplifies the imbalance in the carbon footprint across trade flows.

To date, most research linking international trade with transportation emissions has predominantly focused on maritime trade across various geographical scales (*17*–*19*). However, an exclusive focus on individual transportation modes may overlook the interdependencies and cumulative impacts arising from the collective response of these modes to trade demands. Although some studies have provided insights into international trade-linked CO_2_ emissions by incorporating different transportation modes, the incomplete direct data on these transportation modes has generally led to reliance on fixed continental-averaged modal share data for 2004 (*20*, *21*). While Cristea *et al.* (*20*) have demonstrated that the modal share remains relatively stable across years, no studies have yet confirmed that the heterogeneity of mode share at the regional or commodity scale can be ignored. The choice of transportation mode is influenced by multiple factors, including cost, timeliness, and infrastructure conditions (*22*, *23*), and simplifying the allocation of trade flows to different transportation modes may introduce biases when decoupling CO_2_ emissions in international trade to countries, transport sectors, and commodities. In addition, because of data accessibility limitations, existing studies often use global-averaged carbon intensities for different transportation modes (*20*, *21*, *24*), although these values can vary considerably across different regions and countries due to factors such as fuel type, quality, regulations, and the extent of electrification. As a result, the simplified assumptions regarding transportation modes and carbon intensities in international trade obscure the complexity of trade-linked transportation CO_2_ emissions. While accurate insights can be provided at a macro scale, such simplifications are insufficient for developing targeted decarbonization policies.

**Fig. 1.**
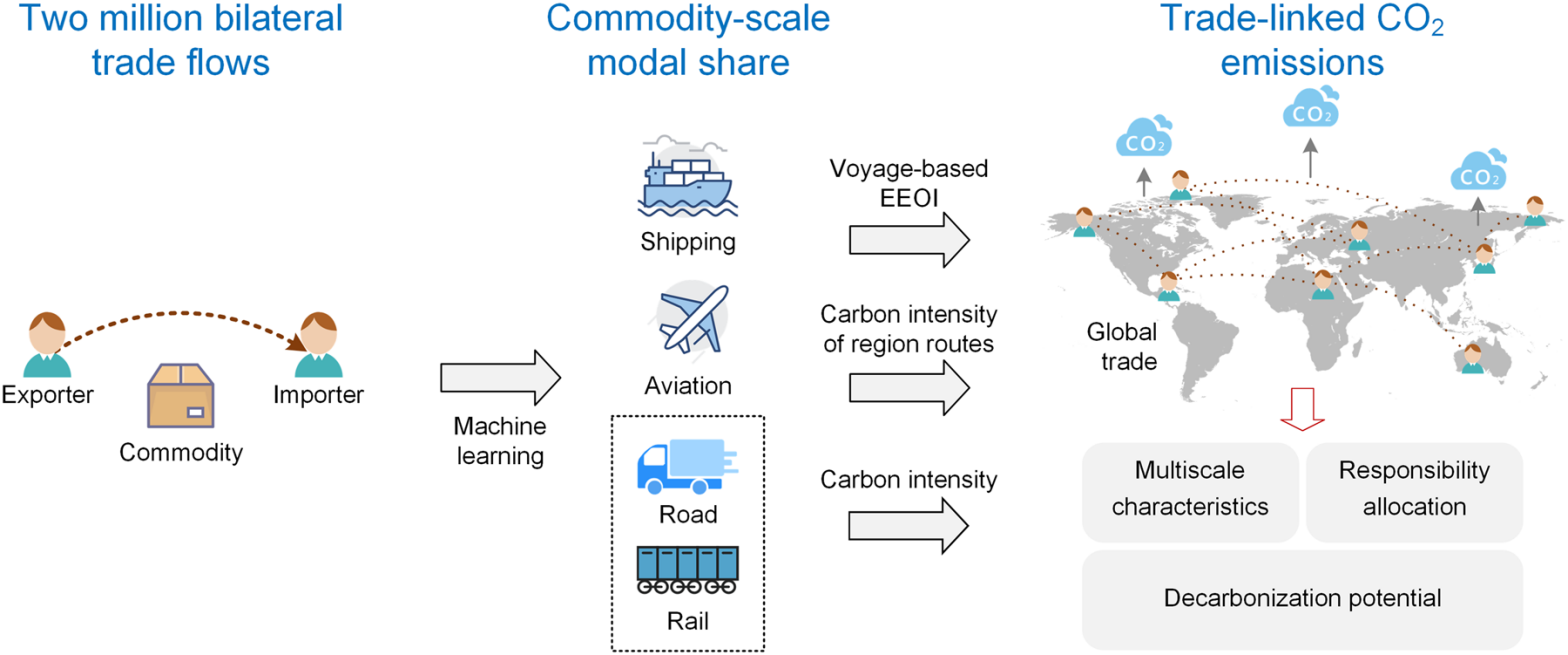
The flowchart of the study design. We developed a machine learning framework to estimate transportation modal share profile across shipping, aviation, road, and rail for each commodity in bilateral trade flows. On the basis of this, we quantified the international trade-linked transportation CO_2_ emissions. The multiscale modal share data reveal the heterogeneity of CO_2_ emissions across scales and highlight the knowledge bias in emission allocation. In addition, we explored the decarbonization potential through trade optimization perspective.

To systematically investigate the international trade-linked transportation CO_2_ emissions, which are defined here as all direct CO_2_ emissions associated with commodities transported internationally via shipping, aviation, roads, and railways, the present study introduces an integrated model linking economics, transportation systems, and emissions models. The integrated model incorporates over 1.9 million trade flows from 65 economies, covering 92% of international trade volume and including 1221 commodities (Fig. 1). This analysis advances the baseline year from the 2010s commonly used in existing studies to the year 2021, and includes the years 1995, 2000, 2008, 2009, and 2019 through 2020 as reference points to investigate historical trends. Considering the diversity of trade pairs and commodities, this study applied an artificial neural network (ANN) to advance the scale of transportation modal share data from the continental scale to the harmonized system (HS) four-digit scale in each trade flow, which represents the finest scale achievable under current publicly available data. To more specifically link emissions with the marine trade, the largest contributor of CO_2_ emissions [accounting for 1.056 billion tonnes in 2018 (*25*)] in international freight transportation, this study replaced the fixed shipping carbon intensity with the vessel energy efficiency operational indicator [EEOI; gCO_2_/tonne per nautical mile (NM)] for each trade pair based on observed fleet activities and Automatic Identification System (AIS) signals. On the basis of the integrated model, this work investigated international trade-linked transportation CO_2_ emissions for different economies, commodities, and transportation modes and analyzed the effects of the modal share on the attribution of CO_2_ emissions. Moreover, this study explored the decarbonization potential through trade optimization from an emerging demand-side perspective. The present study provides an up-to-date perspective on international trade-linked transportation CO_2_ emissions and emissions-reduction potential that have previously received little attention. The detailed framework and quality assurance for the results are explained in Materials and Method.

## RESULTS

### Multiscale transportation modal share profile in international trade

**Fig. 2.**
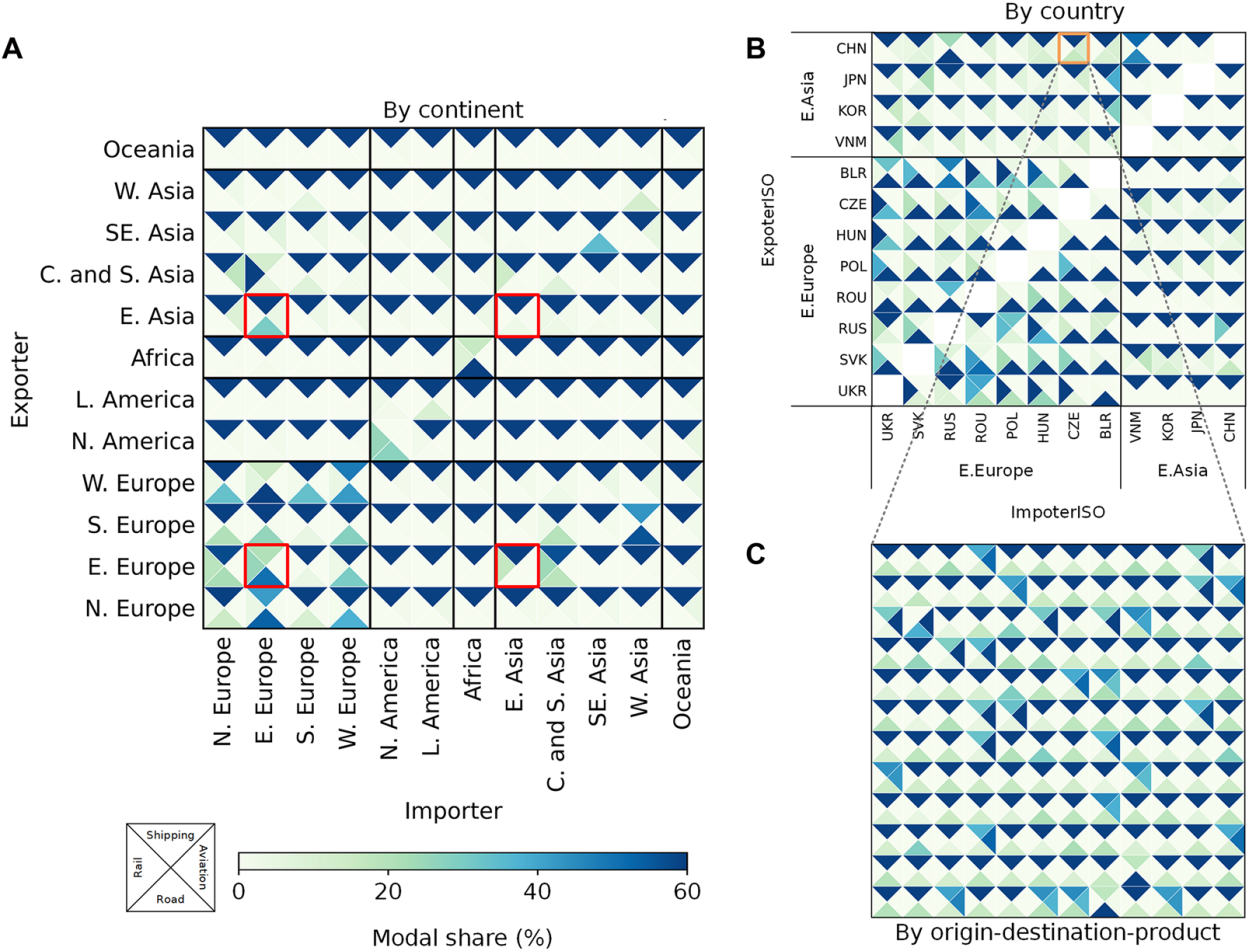
Multiscale transportation modal shares in international trade. (**A**) At the regional scale; (**B**) for countries in East Asia and Eastern Europe; (**C**) for the top 144 traded commodities by weight, based on the harmonized system (HS) four-digit code (arranged from left to right and from bottom to top) in the CHN-CZE pair. Economies are divided into 12 regions following the region groups listed in table S2. The HS four-digit codes for the 144 traded commodities in (C) are listed in table S1. Each entry in the matrix is divided into four triangles, with each triangle representing the emissions contributed by one of the four different transportation modes between pairs of regions.

To more clearly demonstrate the heterogeneity in the choice of transportation modes in international trade, this study first plotted the multiscale transportation modal shares, which were directly calculated based on the average of outputs from the ANN rather than the tonne-km or value. The underlying data used in this study were the transportation modes, namely, shipping, aviation, road, and rail, for each commodity (by HS four-digit code) in each trade pair. The modal share was progressively aggregated from the commodity scale within each trade pair to the trade-pair scale and finally to the regional scale. Figure 2 (A to C) illustrates the decomposition of the modal share from the aggregate to the detailed scale in reverse order. Figure 2A depicts the regional modal share for each origin-destination (OD) flow. Overall, shipping was found to be the primary mode of transportation for international trade across most OD flows, with an average exceeding 90%, although there were notable differences between regions. For example, for European regions with close trading relationships with their land-adjacent economies, the proportion of road transportation ranged from 21 to 77% for export and import activities. For Asia, Africa, and Oceania, 95% of international trade was found to be seaborne. Globally, airborne trade was mainly related to North American trade.

The modal share displayed substantial heterogeneity at both the national and commodity scales within the same region. Figure 2B illustrates the modal share of international trade for countries within and between East Asia and Eastern Europe. For example, in the East Asia–Eastern Europe trade pair, although shipping (65%) and road (30%) transportation accounted for most transportation at the regional scale, island nations such as Japan relied only on shipping and aviation for both imports and exports. For the CHN-VMN trade pair, the proportions of international trade using shipping and roads were nearly equal, which differed considerably from the regional shipping-dominated trend among countries within East Asia. This heterogeneity was even more substantial within the Eastern Europe region.

At the commodity scale, the modal share of the top 144 traded commodities by weight based on the HS four-digit code for the CHN-CZE trade pair is illustrated in Fig. 2C. While a large proportion of commodities were predominantly transported by shipping, which aligned with national-scale statistical patterns, aviation and road transportation also exhibited strong competitiveness for a considerable number of commodities with high trade value, such as chemicals, pharmaceutical preparations, furniture, and artworks (table S1). The heterogeneity in the transportation modal share profile at different scales in global trade highlights the complex nature of global trade. This complexity may also have a substantial impact on international trade-linked transportation CO_2_ emissions accounting, which is further discussed in the following sections.

This study further calculated the share of transportation in tonne-km for 21 commodity categories in the HS section (fig. S1A). Because shipping typically handles the long-distance transportation of bulk commodities, it remained the dominant mode in international trade when measured in tonne-km, accounting for 85%. This was particularly evident in its role in carrying most of the heavy and low-value commodities from sectors such as mineral products (HS section V, 92%) and food products (HS sections I, II, III, and IV, averaging 95%). High-value commodities produced by sectors such as artwork (HS section XXI) and machinery, equipment, and electrical and optical equipment (HS section XVIII) shared 45 to 58% of their transport volume via aviation.

For different transportation modes (fig. S1B), mineral products not classified elsewhere, oil, coal, petroleum, and coal products (HS two-digit code 27) were the primary commodities transported via high-capacity transportation modes, including shipping, road, and rail transportation, especially for rail, followed by ores, slag, and ash (HS two-digit code 26). The results showed that aviation predominantly handled lightweight products, including plastics and articles thereof (HS two-digit code 39) and high-value equipment (HS two-digit codes 84 and 90).

### International trade-linked transportation CO_2_ emissions

**Fig. 3.**
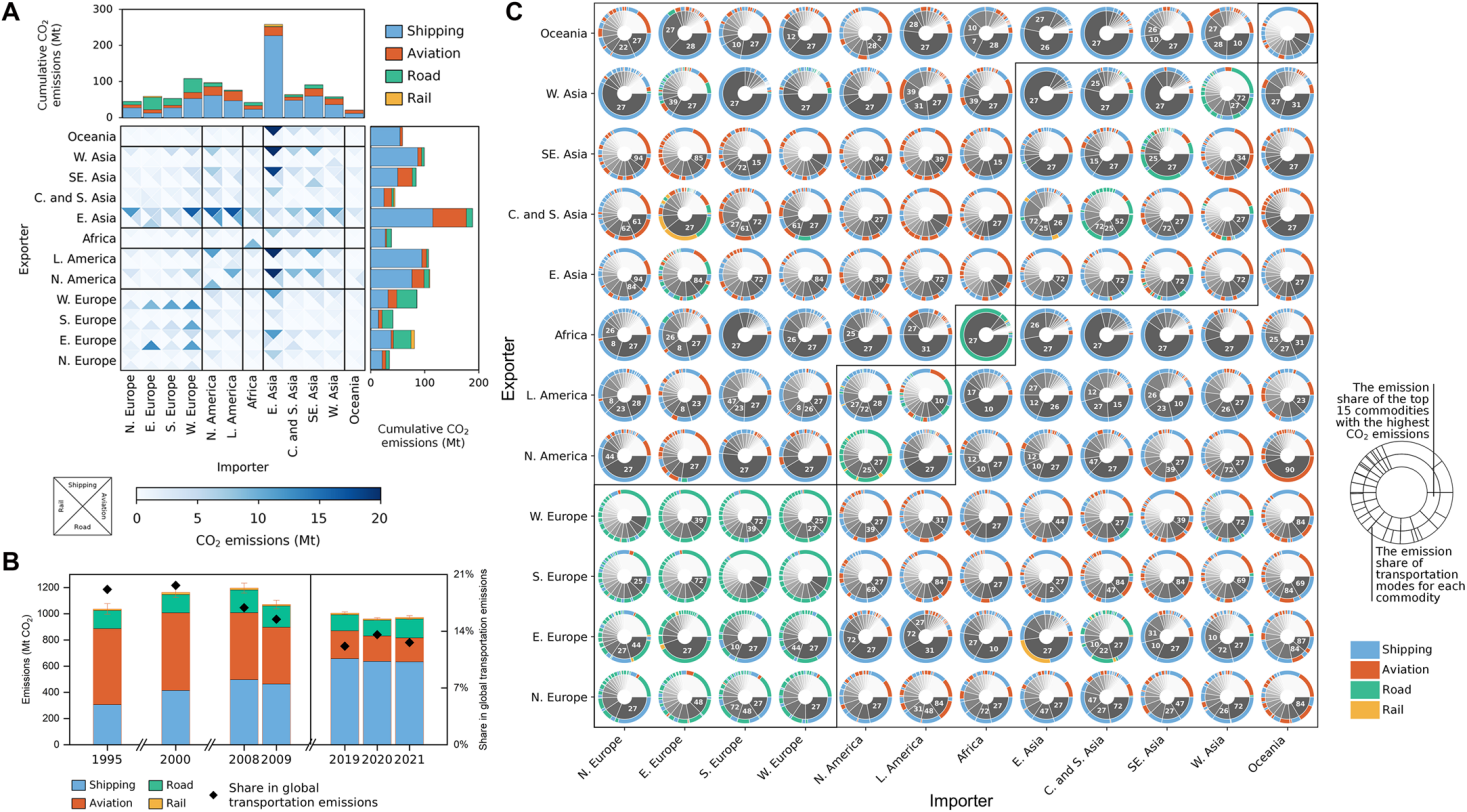
International trade-linked transportation CO_2_ emissions. (**A**) Structure of international trade-linked transportation CO_2_ emissions at the regional scale; (**B**) changes of international trade-linked transportation CO_2_ emissions from 1995–2021; (**C**) decoupling the regional trade-linked transportation CO_2_ emissions produced by commodities and transportation modes. Economies are divided into 12 regions following the region groups listed in table S2. Each entry in the matrix in (A) is divided into four triangles, with each triangle representing the emissions contributed by one of the four different transportation modes between pairs of regions. The pie charts in (C) present the sector code and transportation mode that contribute to the emissions.

Figure 3A illustrates international trade-linked transportation CO_2_ emissions in the latest year, 2021. Each entry in the matrix reveals emissions generated by shipping, aviation, road, and rail transportation for all commodities in each trade pair. It was estimated that international trade in 2021 cumulatively contributed 971 million tonnes (Mt) of transportation CO_2_ emissions, accounting for approximately 12.6% of global transportation CO_2_ emissions, with 62% associated with modal shares supplemented using the ANN. In terms of modal shares, shipping was responsible for most international trade-linked transportation CO_2_ emissions, with 80% of shipping emissions associated with Asia, where the shares of world seaborne trade reached 41 and 61% for commodities loaded and unloaded, respectively (*26*). As the second-largest emitter, aviation accounted for 19% of emissions, notably in trade between East Asia–North America and East Asia–Latin America. Global maps of international trade-linked shipping and aviation emissions are presented in fig. S2. Emissions from road transportation were only found to be substantial for trade within regions and between regions with well-developed and interconnected road networks, such as Europe and North America.

The model developed in the present study traces back to 1995 based on the average transportation modal share results from 2019 to 2021, coinciding with the acceleration of global trade (Fig. 3B). In 1995, international trade-linked transportation CO_2_ emissions amounted to approximately 1016 Mt, representing nearly 19.2% of global transportation CO_2_ emissions that year. The improved fuel efficiency has mitigated the impact of increased trade volume, keeping international trade-linked transportation CO_2_ emissions relatively stable (fig. S3 and table S3). In addition, the share of international trade-linked transportation CO_2_ emissions in global transportation CO_2_ emissions showed a decreasing trend from 1995 (19.2%) to 2021 (12.6%). The contributions of shipping and aviation to emissions displayed a reciprocal trend, with the share of shipping increasing from 30 to 65% and the share of aviation decreasing from 56 to 21%. This change was primarily due to the nearly 200% increase in the transportation work (as defined in Materials and Methods) of shipping during this period, while the transportation work of aviation decreased by nearly half (fig. S4). Details are provided in the “The interannual variation of transportation modes and CO_2_ emissions of global trade” section in the Supplementary Materials.

This study further plotted the top 15 traded commodities with the highest CO_2_ emission shares and their corresponding transportation modes between trading pairs to decouple the international trade-linked transportation CO_2_ emissions produced by commodities and transportation modes (Fig. 3C). Taking bilateral trade among European countries as an example, the traded commodities are chiefly bulk goods. Given the land connectivity between countries, these commodities are predominantly transported via road, especially when Western and Southern European countries act as exporters. In the case of Eastern Europe, such as Russia, a substantial share of bulk commodities is transported via shipping due to the advantages of port access and cost considerations. However, because the shipping distances are generally much longer than those of road transport, the resulting CO_2_ emissions produced by trade with Eastern European exporters are comparable to those involving Western European exporters although shipping has a lower carbon intensity than road freight. Asian countries, as major exporters of high-value manufactured products, exhibit CO_2_ emissions that are predominantly attributed to shipping and aviation, considering the trade-off between cost and timeliness. A typical example can be seen when the United States acts as the importer, where because of the long transportation distance, aviation emissions account for a comparable share to shipping for certain commodities (e.g., HS two-digit code 39) considering the timeliness of transportation via air. In contrast, when Europe is the importer, the shorter distance allows shipping (and road and rail for Eastern Europe) to meet the somewhat reduced timeliness demands, thus greatly reducing the aviation-related emission share. These findings highlight the interactive influence of commodity type, transportation mode, and distance on the carbon footprint of trade flows. This implies that mitigation strategies should address not only technological improvements within modes but also trade structures and the characteristics of traded commodities.

### CO_2_ emissions allocation bias caused by the simplification of modal share

**Fig. 4.**
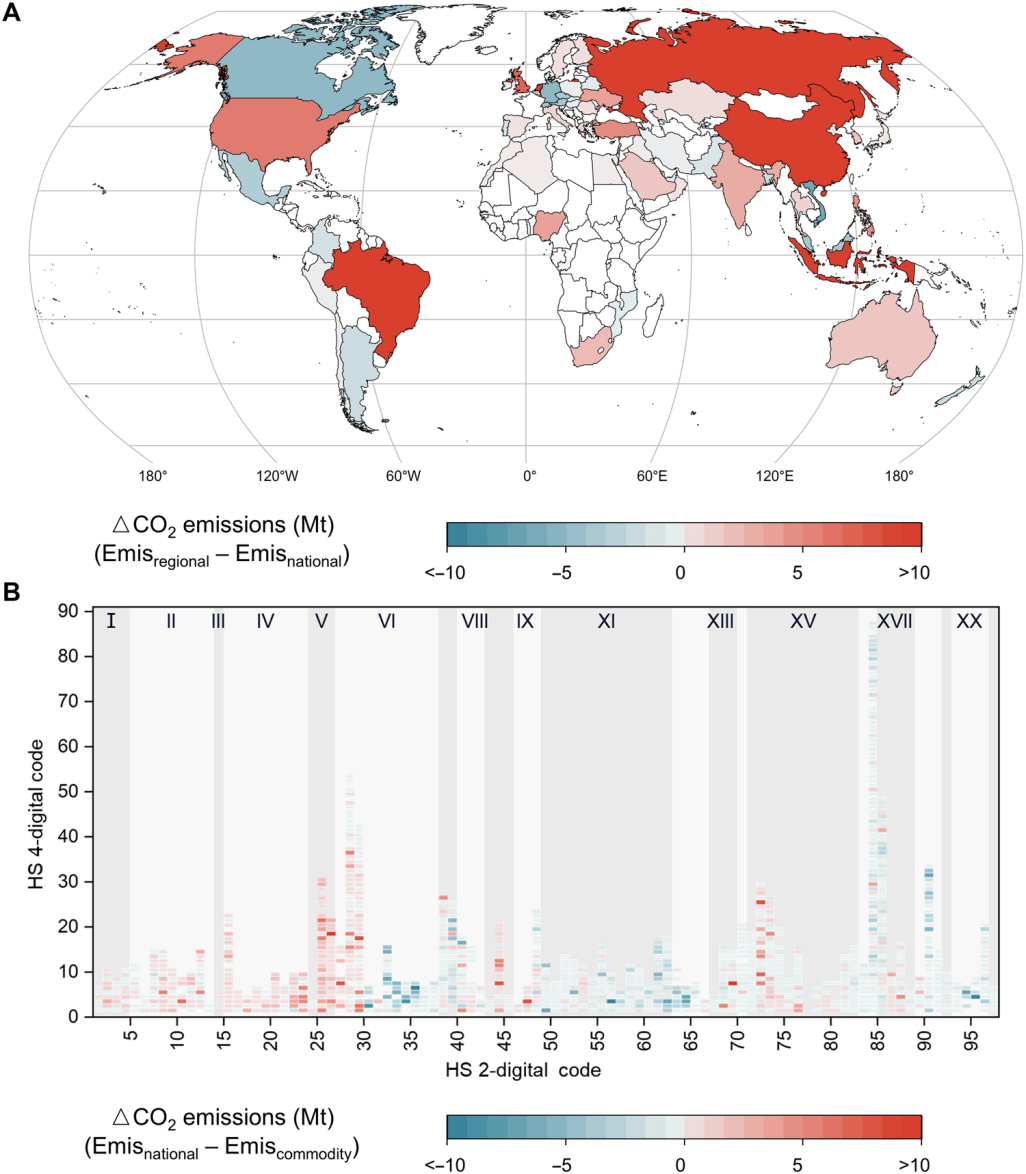
Knowledge bias in international trade-linked transportation CO_2_ emissions based on simplified modal share. (**A**) Differences between national CO_2_ emissions calculated based on regional-scale and national modal shares (continent – national); (**B**) differences between CO_2_ emissions for commodities calculated based on national- and commodity-scale modal shares (national – commodity).

To investigate the impact of the scale of the modal share on international trade-linked transportation emissions estimation, this study further calculated such CO_2_ emissions using regional, national, and commodity-scale modal shares and compared the resulting differences (Fig. 4). While the total CO_2_ emissions estimated based on the commodity-scale modal share (971 Mt) were similar to those estimated using regional and national modal shares (1005 and 975 Mt, respectively), substantial differences were observed across individual countries and commodities.

As shown in Fig. 4A, when regional-scale modal shares were used to estimate emissions at the country scale, the resulting differences ranged from −7 to +17 Mt (with relative deviations reaching up to 140%) compared to those derived from national modal shares. For example, the emissions of countries with a high reliance on low-carbon-intensity transportation modes in international trade, such as China, Brazil, Russia, and Indonesia, tended to be overestimated when regional average modal shares were used, primarily due to the overestimation of the share of high-carbon modes such as aviation. Conversely, for countries heavily dependent on high-carbon modes, such as Canada, where trade with its largest partner, the United States, is dominated by road transport, regional-scale modal shares tended to underestimate the share of road transport and thus underestimate emissions.

Similarly, substantial differences were observed when estimating commodity-specific emissions based on national modal shares instead of commodity-scale modal shares (Fig. 4B). Notably, emissions from bulk commodities that primarily relied on low-carbon transportation modes such as shipping were frequently overestimated, especially for mineral products (HS section V), where emissions were overestimated by 59 Mt (19%). In contrast, emissions from high-value manufactured products were generally underestimated, such as miscellaneous manufactured articles (HS section XX). Moreover, given the substantial differences in carbon intensity across transportation modes (table S3), the allocation bias introduced by simplified modal shares was not merely linearly amplified but rather disproportionately magnified through carbon-intensity weighting. This effect was particularly substantial for countries or commodities with strong reliance on aviation.

It was noteworthy that when emissions were assessed at the national scale, estimates based on national- and commodity-scale modal shares were nearly the same. Similarly, for commodity-scale assessments, national- and regional-scale shares yielded consistent results. In other words, fine-scale modal share data were not strictly required for coarse-scale assessments of aggregate CO_2_ emissions. By contrast, for detailed evaluations at the commodity level, emission heterogeneity was readily obscured if modal shares were simplified. This indicates that matching the resolution of modal share data to the analysis scale is both necessary and sufficient to avoid substantial bias in emissions estimation, as well as ensure a fair allocation of mitigation responsibilities. Otherwise, attribution of emissions to specific countries or commodities may be distorted, which could in turn misguide the design of mitigation strategies. For example, underestimating emissions along road-dominated trade corridors may miss opportunities for short-term reductions through modal shifts or efficiency improvements, while overestimating emissions from bulk commodities may divert attention from high-value product flows with disproportionately high carbon intensities.

### Decarbonization potential of trade optimization

As previously mentioned, the choice of transportation mode and the transportation distance jointly influence international trade-linked transportation CO_2_ emissions. The present study proposes a hypothetical scenario in which each country imports/exports similar commodities (at the HS two-digit scale) to partners with closer route distances, while all other factors remain unchanged to assess the emissions-reduction potential in international trade using the emissions for the year 2021 as the baseline (“Decarbonization potential of trade optimization” section). Because the subcategories within a broad commodity class often differ considerably in terms of characteristics and sourcing requirements, especially for high-value manufactured products, it is not feasible to fully substitute trade partners based solely on shorter transport distances. However, such optimization is potentially attainable for certain raw materials, such as those under HS two-digit code 27. Therefore, this section represents the maximum potential that can be reached through trade optimization.

**Fig. 5.**
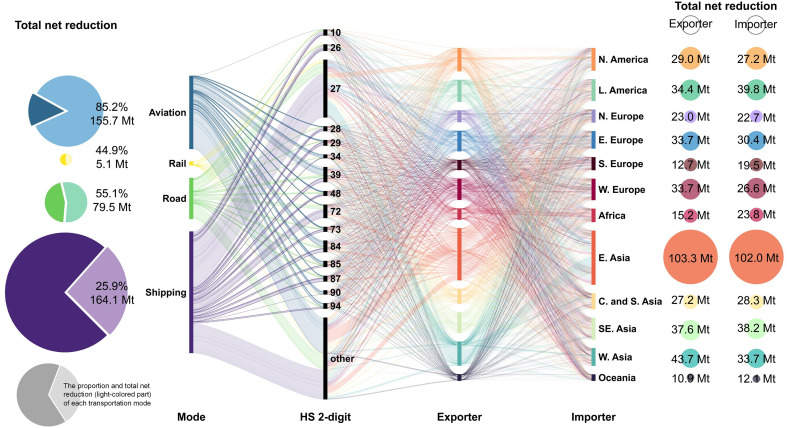
Decarbonization potential of transportation modes, commodities, and regions through trade optimization. The alluvial diagram depicts the net reduction of each mode-commodity-exporter-importer flow. Only the harmonized system (HS) two-digit codes of the top 15 commodities are presented, with the remaining commodities grouped into “other.” The pie charts on the left display the proportion and total net reduction of each transportation mode. The bubbles on the right illustrate the cumulative reduction of each region as an exporter and importer.

In this scenario, international trade-linked transportation CO_2_ emissions can be ideally reduced by 41.6%, equivalent to 404 Mt, with the large volume of emissions reduction associated with shipping (164.1 Mt) and aviation (155.7 Mt). Figure 5 illustrates the decarbonization potential of international freight transportation through trade optimization. For certain raw materials, substantial reductions were observed in mineral fuels (HS two-digit code 27, 94.5 Mt) and iron and steel (HS 72, 21.1 Mt). Because these two categories were primarily transported by sea, shipping contributed to 70.0 and 63.2% of the emission reductions for the mineral fuels category and the iron and steel category, respectively. The decarbonization potential of such raw materials is also reflected in regional trade pairs for which international trade is primarily composed of these commodities. Specifically, the most substantial potential for regional trade emission reductions was found in the West Asia–East Asia flow (27.2 Mt, including 21.7 Mt from HS 27), the Latin America–East Asia flow [23.4 Mt, including 5.8 Mt from HS 27 and 3.4 Mt from HS 47 (wood)], and the East Asia–Latin America flow (19.9 Mt, including 2.2 Mt from HS 72). Further substantial reductions were observed in plastics (HS two-digit code 39, 28.4 Mt), machinery and mechanical appliances (HS two-digit code 84, 21.8 Mt), and electrical machinery and equipment (HS two-digit code 85, 11.3 Mt). This is because these commodities are partly transported by air, which involves many long-distance and high-emission international trade routes. However, the commodity composition at the HS four-digit scale within these categories was highly diverse and the substitutability among commodities was limited, making it difficult to fully realize trade optimization potentials when operating at the HS two-digit scale.

Although this optimization scenario demonstrated substantial decarbonization potential, achieving pure trade-partner optimization in practice would be extremely challenging, given that partner choices are shaped by multiple factors, including geopolitics (e.g., wars, sanctions) (*27*, *28*), economics (e.g., climate policies, trade disputes) (*29*, *30*), and technological constraints (*31*, *32*). Nevertheless, this assessment highlights that countries could shift their decarbonization focus from the end (emissions) to the start (demand), since end-of-pipe controls often require long cycles of technological advancement. In addition, it must be acknowledged that such a transition also requires countries to take emission reduction as part of their own development strategies and objectives proactively rather than passively.

## DISCUSSION

In 2021, international trade-linked transportation contributed to 971 Mt CO_2_ emissions, representing a 6.3% decrease despite the overall rising transportation work between 1995 and 2021. This reduction was primarily due to collective efforts in fuel optimization across the four main transportation sectors. In addition, a gradual shift in transportation modes toward lower-carbon patterns, such as shipping, has also played a role in reducing emissions.

It should be noted that the modal share in global trade transportation exhibited substantial heterogeneity across different scales. This suggests that attributing CO_2_ emissions to individual countries or commodities requires the use of modal share data that corresponds to the same scale. International trade-linked transportation CO_2_ emissions are a direct result of the interplay among commodity characteristics, transportation modes, and geographical distance. This complexity necessitates decarbonization strategies that simultaneously address modal shifts, fuel efficiency improvement, and supply chain restructuring. Given the resilience of transportation patterns to economic shocks and the COVID-19, as well as the long timescales required for technological advancements, the first two goals are inherently incremental processes, making the path to net-zero emissions highly challenging.

Therefore, shifting from end-of-pipe emission control to upstream demand optimization shows a remarkable potential for reducing trade-linked transportation emissions. Nevertheless, it is essential to acknowledge that the optimization process developed in this study does not account for real-world constraints such as geopolitics, economics, and technological limitations, all of which can substantially affect trade structures. For instance, geopolitical disruptions may cause short-term shifts in trade flows. A recent example is the Russia-Ukraine conflict, where restricted access to Black Sea ports and a series of sanctions forced widespread rerouting of trade. In response, countries such as Oman and Saudi Arabia increased wheat imports from existing partners (e.g., Australia to Oman) and new partners (e.g., India to Oman) (*28*). This case suggests that in the face of sudden disruptions, staple commodities with relatively mature markets and well-established supply chains are more amenable to substitution, highlighting a promising entry point for decarbonization through trade optimization in essential bulk commodities. In addition, although maritime and air transport were disrupted, the COVID-19 pandemic accelerated the growth and importance of trans-Eurasian rail transport, which is promoting regional trade development and giving rise to new trade relationships. This efficient and cost-effective mode of transport also offers a new perspective for trade optimization. However, such transitions are not without trade-offs. For example, Global South exporters of bulk commodities may face reduced competitiveness or shifting demand patterns if trade reconfiguration favors geographically closer partners. This raises concerns over equity and development opportunities in regions already facing structural disadvantages. Furthermore, entrenched geopolitical alignments, trade agreements, and infrastructure dependencies may limit the feasibility of rerouting trade purely on the basis of emissions efficiency. As such, any future implementation of trade optimization must be sensitive to these distributional and geopolitical constraints to ensure a fair and realistic transition.

In contrast, the impact of long-term structural drivers such as economics is less immediate but more enduring. For instance, climate policies (e.g., the European Union Emissions Trading System and the Carbon Border Adjustment Mechanism) assign a monetary value to a company’s GHG emissions, thereby directly altering the relative competitiveness of trading partners and routes (*33*, *34*). Such measures can also complement end-of-pipe emission control by creating sustained incentives for technological innovation, such as the adoption of alternative fuels. Furthermore, the uneven global distribution of alternative fuels, advances in transportation efficiency, and coordinated logistics planning influence which trade partners can reliably support sustained low-carbon transport. These developments suggest that structural decarbonization and end-of-pipe measures should not be viewed as an either-or proposition but rather as mutually reinforcing strategies. Therefore, future trade optimization frameworks could incorporate both short-term disruptions and long-term structural dynamics, while balancing system-level decarbonization and end-of-pipe controls based on factors such as cost-effectiveness, technological readiness, and regional fuel availability. Such integration enables more practical and implementable decarbonization pathways.

## MATERIALS AND METHODS

### Technical framework and data sources

In this study, we constructed an integrated model to systematically investigate trade-linked transportation (shipping, aviation, road, and rail) emissions of global trade flows, with an emphasis on 2021. In addition, we extended the study period to include some earlier years, specifically 1995, 2000, 2008, 2009, 2019, and 2020, to focus on historical evolutionary patterns of trade-linked transportation. The year 1995 was chosen because this is the earliest available dataset, serving as a baseline for understanding long-term trends. The year 2000 represents a crucial point at the turn of the 21st century. The inclusion of the years 2008 and 2009 aimed to comprehend the impacts of the global economic recession during that period. The years 2019 and 2020 were selected to study the impact of the COVID-19. The model’s structure is depicted in fig. S5, and can be divided into three major modules: trade data processing, transportation modal share prediction, and emissions calculation.

Here, international trade data were collected from the Base pour l’Analyse du Commerce International (BACI) database developed by Le Centre d’études prospectives et d’informations internationales (CEPII; http://www.cepii.fr). This database includes bilateral trade value (in thousand USD) and volume (in tonnes) for more than 5000 commodities from over 200 economies. Following the filtering principles outlined in the “Trade data filtering” section in the Supplementary Materials, approximately 1.8 to 2.0 million trade flows were screened out each year. The trade structure aggregated at regional scale for the 2021 is shown in fig. S6 as an example.

We further collected the transportation modal share data for the years 2019 to 2021 from the United Nations Comtrade database (https://comtrade.un.org/), the Eurostat database (https://ec.europa.eu/eurostat/data/database), and the US census (https://usatrade.census.gov). We processed data cleaning by removing unreasonable land transportation records in trade between islands and air transportation records in high weight-to-value ratio commodities, such as minerals and crude oil. The aforementioned databases can provide transportation modal share information for 52 to 58% of our trade flow each year. To fill the missing modal share data, we used machine learning, specifically selecting the ANN after extensive review and testing, as detailed in the “Modal share estimation” section in the Supplementary Materials. It is important to note that, for years before 2019, we applied the average transportation modal share results from 2019 to 2021 (fig. S7), considering the results before 2019 for reference only.

In emissions calculation module, we integrated VoySEIM (Voyage-based Shipping Emission Inventory Model), developed by our team, to estimate input variables for shipping, including shipping distance, EEOI, and vessel type. VoySEIM is capable of estimating the annual EEOI of a certain vessel type occurring between the departure and arrival countries. The detailed process has been discussed in the “Energy efficiency operational indicator” section. Bilateral distance used in road and railway emission is the great circle distance (GCD) derived from the CEPII Geodist database (https://cepii.fr/CEPII/en/bdd_modele/bdd_modele_item.asp?id=6). The emission intensity for road and railway is 112.76 and 14.08 gCO_2_/tonne-km, respectively. For the aviation emissions, we corrected the GCD according to factors provided by the ICAO to produce air transport distance. The aviation emission intensity is our calculation based on the International Council of Clean Transportation’s (ICCT) report (*35*) (“The aviation CO_2_ emissions intensity” section in the Supplementary Materials). The selected emission intensity of all target years is provided in table S3.

### International trade-linked transportation emissions calculation

Once all the components are set, the model is computed sequentially. The emission calculation formula for aviation, road, and rail is

$$E_{o,d,g,m}=\sum_{m}W_{o,d,g}\times {\mathrm{MS}}_{o,d,g,m}\times {\text{DIST}}_{o,d,m}\times e_{m}\times 10^{-6}$$

Denote $E_{o,d,g,m}$ as the CO_2_ emission produced by aviation, road, and rail in a single trade flow (exporter *o* transports commodity *g* to importer *d*), in tonnes. $W_{o,d,g}$ is the weight of trade flow in tonnes. A trading pair can transport the commodity by using different transportation modes. The ${\text{MS}}_{o,d,g,m}$ represents the share of mode *m*, so $W_{o,d,g}\times {\text{MS}}_{o,d,g,m}$ gives the weight moved by mode *m* in the trade flow, in tonnes. ${\text{DIST}}_{o,d,m}$ refers to the distance traveled from *o* to *d* by mode *m*, in km. The value of ${\text{DIST}}_{o,d,m}$ depends on the mode *m*. For road and rail, we used GCD data as travel distance. While for aviation, we adopted the GCD correction factor released by ICAO to calculate the travel distance. After multiplying ${\text{DIST}}_{o,d,m}$, we can get the transportation work of each mode, measured in tonne-km. By multiplying $e_{m}$, the CO_2_ emission intensity (in gCO_2_/tonne-km) of mode *m* to get the CO_2_ emission produced by mode *m*, in grams. Last, sum the emission of different modes *m*, to get the CO_2_ emission of aviation, road, and rail.

The emission calculation for maritime transportation is based on the following equation

$$E_{o,d,g,s}=\sum_{v}W_{o,d,g}\times {\text{MS}}_{o,d,g,s}\times {\text{VD}}_{g,v}\times {\text{DIST}}_{o,d,s}\times {\text{EEOI}}_{o,d,v}\times 10^{-6}$$

Denote $E_{o,d,g,s}$ as the shipping CO_2_ emission produced by a single trade flow (exporter *o* transports commodity *g* to importer *d*, *s* represents shipping), in tonnes. $W_{o,d,g}$ is the weight of trade flow in tonnes. The ${\text{MS}}_{o,d,g,s}$ represents the share of shipping in the trade flow. ${\text{VD}}_{g,v}$ is the vessel distribution coefficient of commodity *g* transported by vessel type *v*. ${\text{DIST}}_{o,d,s}$ is the shipping distance between exporter *o* and importer *d*, in NM. ${\text{EEOI}}_{o,d,v}$ is the EEOI for vessel type *v* voyaging from exporter *o* to importer *d*, in gCO_2_/tonne-NM, with details described in the next section. So, $W_{o,d,g}\times {\text{MS}}_{o,d,g,s}\times {\text{VD}}_{g,v}\times {\text{DIST}}_{o,d,s}\times {\text{EEOI}}_{o,d,v}$ gives us the CO_2_ emissions of that trade flow using vessel type *v*. Sum the emissions of all types of vessels yield the shipping emissions of that trade flow. Details for identifying the shipping voyage are described in the next section.

Last, add the emissions of aviation, road, and rail with shipping to get the transportation CO_2_ emissions contributed by a single trade flow.

$$E_{o,d,g}=E_{o,d,g,m}+E_{o,d,g,s}$$

While presenting the results, we aggregated the 65 economies into 12 regions, according to the region group in table S2.

### Energy efficiency operational indicator

EEOI (in gCO_2_/tonneNM) differs from conventional emission factors (in g/km or g/kg) as it links shipping emissions with trade volumes. The calculation of EEOI follows three steps (fig. S8): First, (a) the international voyage is identified according to the actual shipping routes between departure and arrival countries based on AIS data. Next, (b) shipping emissions is estimated on the basis of the time interval between two consecutive AIS signals using the vessel’s instantaneous engine power and power-based emission factors, and then aggregated for each voyage [the technical details of steps (a) and (b) are provided in the “International voyage identification and emission calculation” section in the Supplementary Materials]. Last, (c) the voyage-based EEOI can be calculated using equation

$${\text{EEOI}}_{v,b,i}=\frac{E_{v,b,i}\times \left(1+{\text{BAF}}_{v,b}\right)}{{\text{DWT}}_{i}\times {\text{TCU}}_{v,b}\times D_{v,b,i}}\times 10^{6}$$

where ${\text{EEOI}}_{v,b,i}$ is the EEOI for voyage i, classified by vessel type v and size bin b, in gCO_2_/tonne-NM; $E_{v,b,i}$ is the shipping CO_2_ emissions for voyage i, classified by vessel type v and size bin b, in tonnes; ${\text{BAF}}_{v,b}$ is the berthing adjust factor for vessel type v and size bin b (unitless); ${\text{DWT}}_{i}$ is the vessel’s dead weight tonnage for each voyage i, in tonnes; ${\text{TCU}}_{v,b}$ is the total capacity utilization for type v and size bin b (unitless); and $D_{v,b,i}$ is the shipping distance for voyage i, classified by vessel type v and size bin b, in NM.

Next, the ${\text{EEOI}}_{v,b,i}$ are averaged by vessel type and the *o*-*d* country pair, as shown by equation below

$${\text{EEOI}}_{o,d,v}=\frac{\sum_{b}\sum_{i}\left[E_{v,b,o,d,i}\times \left(1+{\text{BAF}}_{v,b}\right)\right]}{\sum_{b}\sum_{i}\left({\text{DWT}}_{i}\times {\text{TCU}}_{v,b}\times D_{v,b,o,d,i}\right)}\times 10^{6}\left(o\neq d\right)$$

where $E_{v,b,o,d,i}$ is the shipping CO_2_ emissions for voyage i between country o and country d, classified by vessel type v and size bin b, in tonnes; $D_{v,b,o,d,i}$ is the shipping distance for voyage i between country o and country d, classified by vessel type v and size bin b, in NM.

### Decarbonization potential of trade optimization

To explore the potential of trade optimization on international freight transportation while ensuring the current international trade volume, we extend model of shipping decarbonization (*19*) potential to all international transportation modes. When each country imports/exports similar commodities to partners with closer route distance and or chooses routes with better energy efficiency, it is possible to achieve transportation emissions reduction. This scenario can be modeled as a linear programming problem. The objective function in this model aims to minimize the total emissions from international transportation. The constraints ensure that the total import and export volumes (by weight) of each country’s international trade remain constant, ensuring that the trade flows do not exceed any country’s production capacities and demands. It is essential to note that certain factors, such as transportation costs, international relations, and trade agreements, are not considered in this analysis. Consequently, the results presented here represent the maximum potential for decarbonization.

A linear programming model is developed with a single objective of minimizing the total emissions of international freight transportation for the year 2021. The constraints ensure that the total import and export volumes of each economy remain constant. The optimization is performed at the HS four-digit commodity scale, utilizing the following equations

$$E_{\text{min}}=\sum_{g}E_{\text{min},g}$$

$$\begin{array}{c} E_{\text{min},g}=\sum_{o,d}W_{o,d,g}^{\prime }\times \left(m_{s,o,d,g}+m_{a,o,d,g}+m_{\mathrm{ra},o,d,g}+m_{\mathrm{ro},o,d,g}\right)\times 10^{-12}=\sum_{o,d}W_{o,d,g}^{\prime }\\ \times \left[\left(\sum_{v}{\text{VD}}_{v,g}\times {\text{EEOI}}_{o,d,v}\times {\text{DIST}}_{o,d,s}\times {\text{MS}}_{o,d,g,s}\right)+\left(e_{o,d,a}\times {\text{DIST}}_{o,d,a}\times {\text{MS}}_{o,d,g,a}\right)\right.\\ \left.+\left(14.08\times {\text{DIST}}_{o,d}\times {\text{MS}}_{o,d,g,\mathrm{ra}}\right)+\left(112.76\times {\text{DIST}}_{o,d}\times {\text{MS}}_{o,d,g,\mathrm{ro}}\right)\right]\times 10^{-12} \end{array}$$

$$\text{s.t.}\left\{ \begin{array}{c} \sum_{d}W_{o,d,g}^{\prime }\leq \sum_{d}W_{o,d,g},\left(o=1,2,3\ldots 65\right)\\ \sum_{o}W_{o,d,g}^{\prime }\leq \sum_{o}W_{o,d,g},\left(d=1,2,3\ldots 65\right)\\ \sum_{o,d}W_{o,d,g}^{\prime }=\sum_{o,d}W_{o,d,g}\\ W_{o,d,g}^{\prime }=0\left(o=d\right)\\ W_{o,d,g}^{\prime }\geq 0\left(o\neq d\right) \end{array}\right.$$

Denote $E_{\text{min}}$ represents the total emissions of international freight CO_2_ transportation, in Mt. $W_{o,d,g}^{\prime }$ and $W_{o,d,g}$ are the optimal and original trade volumes (in tonnes) of commodity *g* between exporter *o* and importer *d*, respectively. ${\text{VD}}_{v,g}$ is the vessel distribution coefficient of commodity *g* transported by vessel type *v*. ${\text{EEOI}}_{o,d,v}$ is the EEOI for vessel type *v* voyaging from exporter *o* to importer *d*, in gCO_2_/tonne-NM. ${\text{DIST}}_{o,d,s}$, ${\text{DIST}}_{o,d,a}$, and ${\text{DIST}}_{o,d}$ is the shipping distance (in NM), aviation distance (in km), and GCD (in km) between exporter *o* and importer *d*, respectively. ${\text{MS}}_{o,d,g}$ represents the modal share proportion (%) of shipping *s*, aviation *a*, railway *ra*, and road *ro*. $e_{o,d,a}$ is the CO_2_ emission intensity of aviation for commodity transported from exporter *o* to importer *d*.

### Quality assurance

Throughout model development, the intermediate parameters and final results have been validated by quality assurance and control and compared with external sources to minimize uncertainties, as follows: First, when integrating VoySEIM, a series of filters were applied to reduce the deviation of international voyage identification and EEOI estimation caused by unreliable ship data and voyages, details were presented in our previous study. In addition, to focus on major commodities and economies and to reduce uncertainties in the base data, we filtered and optimized global trade data from BACI, and missing data were supplemented. Details are provided in the “Trade data filtering” section in the Supplementary Materials.

Second, modal shares are one of the important intermediate parameters in this study. We have conducted both internal and external validations for the predicted modal shares. For internal validation, we used 10-fold cross-validation while building the ANN model, verifying the model’s robustness through the calculation of mean square error, correlation coefficient, and classification accuracy for the most predominant mode. For external validation, we compared the average predicted modal shares from 2019 to 2021 at both regional and national scales with those from other studies. Detailed descriptions of the modal shares validation are provided in the “Modal share estimation” section in the Supplementary Materials.

Third, the international trade-linked transportation emissions results were compared with external work for verification. At the global scale, the total international shipping and aviation emissions were verified by comparing with results from organizations, including the IEA, IMO, and ICCT, as well as two global-scale studies (*20*, *21*). At the finer scale, the total trade-linked transportation emissions in 2019 for 10 countries were also well verified by comparing with a previous study (*21*). The related results are displayed in fig. S9. For the sector with the largest emissions, shipping, we also analyzed the differences in the comparisons, with some evidence presented in fig. S10. Detailed descriptions are provided in the “CO_2_ emissions validation” section in the Supplementary Materials.

### Uncertainty analysis

This study recognized the major uncertainties in the model.

#### Assumptions

1) The transportation modal share remains the same (average transportation modal share results from 2019 to 2021) for the years before 2019. Therefore, the results before 2019 are only used for comparison and reference. It is worth noting that this assumption has minimal impact on the inter-annual variation of emissions (*20*).

2) The distance used in the emissions calculations of aviation, road, and railway was not the actual transportation distance, but follows GCD or corrected GCD (*20*, *21*).

3) The emission intensities for aviation, rail and road remain the same for 2019–2021.

#### Uncertainties

First, this study uses ANN to supplement the missing modal share data. In reality, the choice of transportation modes is determined by multiple stakeholders and factors, such as freight charges and country relations (*36*). As this study is unable to incorporate all of these variables into the model to present an exact real-world modal-share profile, which leads to prediction errors and introduces uncertainties. Next, ANN essentially functions as a “black-box” prediction model during the inference process, where the decision-making process is not analytically traceable. Although sensitivity analyses can help assess the impact of certain variables, it remains difficult to explain the logic behind the modal choice for specific trade pairs. Last, although cross-validation demonstrates that the ANN model performs robustly overall, its predictions should be interpreted with caution for trade pairs characterized by sparse data or high heterogeneity. While we did not observe systematic error patterns across these cases, such limitations are commonly encountered in data-driven approaches. Therefore, whether interpreting ANN-derived modal shares or assessing the influence of specific input variables, we recommend supporting such interpretations with external evidence such as independent datasets, prior studies, or mechanistic simulations. These complementary sources are essential for validating both the model’s outputs and the underlying mechanisms that cannot be fully explained by the model alone.

Second, the exclusion of GCD correction factors for land-based transport, such as road and rail, may introduce uncertainties into our emissions estimates. Specifically, in industrialized countries, road-based transport typically has correction factors ranging from 1.3 to 1.6, while rail transport may require even higher correction factors due to the sparser track infrastructure. Without these corrections would lead the underestimation of emissions for land-based transportation modes.

Third, a unified carbon intensity sourced from the EU and the USA was applied for trucks and trains. The actual carbon intensity of land transportation in less-developed countries might be higher due to differences in infrastructure, vehicle fleet composition, technological standards, and regulatory frameworks. This discrepancy could introduce uncertainties in emissions calculations, particularly in regions with less developed transportation systems where older and less efficient vehicles dominate, or where regulations on emissions are less stringent. For instance, outdated vehicle technologies and poorly maintained road infrastructure could lead to higher fuel consumption per tonne-km, increasing the carbon intensity of transportation.

## Supplementary Materials

### This PDF file includes:

Supplementary Text Figs. S1 to S11 Tables S1 to S10 References
