# Supplementary material for: Global mapping of disaggregated international trade-linked transportation CO2 emissions
Source: Sci Adv. 2026 Apr 3;12(14):eadz1670. doi: 10.1126/sciadv.adz1670 (PMC13048254; doi:10.1126/sciadv.adz1670)
Supplement: Supplementary file 1 — Supplementary Text Figs. S1 to S11 Tables S1 to S10 References [file sciadv.adz1670_sm.pdf]

Science Advances
AAAS

Supplementary Materials for

Global mapping of disaggregated international trade-linked transportation CO_2 emissions

Zhenyu Luo *et al.*

Corresponding author: Huan Liu, liu_env@tsinghua.edu.cn

Sci. Adv. **12**, eadz1670 (2026)
DOI: 10.1126/sciadv.adz1670

This PDF file includes:

Supplementary Text
Figs. S1 to S11
Tables S1 to S10
References

Supplementary Text

Trade data filtering

Trade data for this study are obtained from Le Centre d'études prospectives et d'informations internationales (CEPII; http://www.cepii.fr). They develop the Base pour l'Analyse du Commerce International (BACI) database by aggregating and standardizing data from various customs records and trade statistics. The BACI database categorizes commodities according to the Harmonized System (HS) code subheadings (HS 6-digit). HS is developed by the World Customs Organization (WCO), commonly used by the over 200 countries and economies in customs tariffs and international trade data (*37*) .To better focus on major commodities and economies, we proceed with the following steps to narrow our study scope:

a) Merge the original HS 6-digit data into HS four-digit.
b) Exclude the data of electric energy (HS 2716) from the database, as it does not rely on transportation modes we studied.
c) Rank economies by GDP (collected from the World Bank: https://data.worldbank.org/indicator/NY.GDP.MKTP.CD), trade values, and trade weight in year 2019, 2020, and 2021. The trade value and weight are the sums of exports and imports in BACI.
d) Select the top 50 economies of each indicator each year.

Step a and b result in 1221 types of commodities, and steps c and d screen out 65 economies. On average, these economies accounted for more than 96% of global GDP, 94% of global trade value, and 92% of global trade volume between 2019 to 2021. The ranking and data sources of economy selection are provided in **table S2**. An overview of the trade flow profile is given in **table S4**.

Regarding missing volume in BACI, we calculate the volume of this trade flow by multiplying the average weight-to-value (WV) ratio (in tonnes/thousand USD) of this commodity exported by the corresponding exporter with its trade value. If data about the exporter are not available in the original database, we calculate the volume using the global average WV ratio of that commodity. **Fig. S7** gives the structure of trade volume data in the model, aggregated at regional level.

Modal share estimation

The traditional approach to studying the international freight transportation system largely categorized into statistical modeling and operations research (*38*). Driven by advancements in computing power and big data analytics, machine learning approaches have gained attention in freight transportation mode research over the last decade.

Considering the ability for multi-target prediction and the generalization capability for samples beyond the range of the training data, Artificial Neural Network (ANN) is more suitable for predicting transportation modal share in this study. Additionally, we find that ANN is the most popular machine learning methods for predicting commodity demand in shipping and aviation (*37*). The earliest use of ANN in demand forecasting can be traced back to Wei et al. (*39*) who predicted the throughput of transshipment containers at Kaohsiung port, Taiwan, using ANN. Lam et al. (*40*) demonstrated that ANN can provide more accurate results than regression models in forecasting freight movements in ports. Container flows between multiple Asian ports were successfully presented by Tsai and Huang (*41*). In terms of air cargo, Chen et al. (*42*) made the first attempt to estimate the supply from Japan to Taiwan, while Loaiza et al.

(*43*) later applied ANN in predicting air cargo flows within Columbia. For intermodal transportation, Bilegan et al. (*44*) employed ANN to forecast freight demand at Canadian intermodal terminals. Wu and Liu (*45*) forecasted the capacity growth of sea-rail intermodal transportation at Ningbo port, China, while Abdirassilov and Sładkowski (*46*) targeted the China-European corridor and showed that a wider geographic scope can be examined using ANN.

As the development of machine learning depends on the data availability and integrity of inputs, adequate attention must be paid to the sources. Most studies considered import and export volume, GDP, population, commodity types, inflation rates, and fuel prices (*39-41, 43, 47-49*). Research specific to certain port or country maritime demand also included Automatic Identification System (AIS) data or container throughput (*50-52*).

When performing multi-targets regression using ANN, all trading pairs are divided into two datasets based on geographical location for better estimation results. The first one is the "Land" dataset, consisting of trading pairs that are connected by land and can move freight by using shipping, aviation, road, and railway. The other trading pairs belong to the "No Land" dataset, consisting of trading pairs that are not connected by land and can move freight by using only shipping and aviation. The input variables in "Land" and "No Land" datasets are almost the same, including:

a) Exporter and importer's GDP in the corresponding year
b) Bilateral distance: great circle distance
c) Commodity category: HS Code chapter (two-digit) and heading (four-digit)
d) Trade value and volume in the corresponding year
e) Exporter-commodity-based weight-to-value ratio
f) Infrastructure quality of airport, road, railway, and port, for both exporter and importer
g) Geographical group: Island, North America, South America, Africa, Southeast Asia, West Asia, Rest of Asia, and Europe
h) Landlocked country: dummy variable
i) Land contiguous: dummy variable
j) Common official languages: dummy variable
k) FTA: dummy variable

The predicted variables in "Land" include the modal share proportion of shipping, aviation, road, and railway, and the predicted variables in "Land" include the modal share proportion of shipping and aviation.

GDP is collected from The World Bank (https://data.worldbank.org/indicator/NY.GDP.MKTP.CD). The great circle distance between economies and dummy variables indicating whether they are landlocked, contiguous, share a common official language, are derived from the CEPII Geodist database (https://www.cepii.fr/CEPII/en/bdd_modele/bdd_modele_item.asp?id=6). The trade value and volume are collected from the Base pour l'Analyse du Commerce International (BACI) database developed by Le Centre d'études prospectives et d'informations internationales (CEPII, http://www.cepii.fr). The dummy variable representing bilateral or plurilateral Free Trade Agreements is based on the list published by Asian Development Bank (https://aric.adb.org/fta-country) and Wikipedia (https://en.wikipedia.org/wiki/List_of_bilateral_free-trade_agreements). We use World

Economic Forum's assessment on transportation mode infrastructure quality to express the impacts of infrastructure on mode choice (https://www.theglobaleconomy.com/rankings/seaports_quality/).

We conduct several rounds of tests to determine suitable hyperparameters, including learning rate, activation function, normalization method, the number of hidden layers, and the number of neuron nodes in the hidden layers. Regarding the number of neuron nodes in hidden layers, many empirical formulas and rules exist to determine the number; we follow the rule that the number of hidden neurons should be between the size of the input layer and the size of the output layer (*53*). The hyperparameter settings are given in **table S5**. All training sessions end because of the loss on the validation set does not decrease for 6 consecutive epochs. Additionally, due to the train-test split evaluation proportion cedure, the model will generate different results even running the same dataset and algorithm. This study trains each dataset 10 times to investigate the potential differences brought by the evaluation procedure in Matlab R2022b separately.

Here, the 10-fold cross-validation repeated 10 times is considered to evaluate the prediction performance of our models. The total dataset was randomly divided into 10 subsets, where 9 subsets was used to train the model and another was applied for validation. After training, the mean square error (MSE) and correlation coefficient (R) are calculated to evaluate the simulation results of ANN. Since there are no significant differences between each training, the simulation results that yield the highest maritime volume are selected. In addition, we calculate the classification accuracy (CA) for the most predominant mode of transportation, as this mode has the greatest impact on emissions. The prediction performance of the chosen results is provided in **table S6**.

Considering that not all influencing factors of transportation mode choice could be included in the ANN, we perform an optimization, especially on the transportation modal share of high weight-to-value ratio commodities, such as cereals, mineral products, and stone. The above optimization steps successfully increase the R of commodity transported by ships to more than 0.95, when applied to statistical data, proving the rationality and logic of the optimization steps.

Here, we perform an external validation of the modal share for different transportation modes by comparing them with other studies at both regional and national scales. The regional modal share results, averaged from 2019 to 2021, are compared with the results from 2004 in **table S7**. The national modal share results are compared with the results from 2015 in **table S8**. The average absolute errors in modal shares by exporter are14% for shipping, 11% for aviation, and 18% for land transportation. For modal shares by importer, the average absolute errors are 15%, 10%, and 21%, respectively. Overall, our prediction is well, with the acceptable differences compare with previous studies. The differences primarily arise from different target years and calculation methods. This demonstrates the robustness of our predicted modal share.

This study also assesses the impact of input variables on output variables, i.e., parameter sensitivity, by comparing the changes in output variables after modifying input variables. Trade data from the year 2021 is employed for this evaluation. In the evaluation process, each input dataset undergoes a 10% increment or decrement in one dimension while keeping other dimensions constant. The modified input dataset is then fed into the trained neural network to obtain new predictions, which are compared with the original values. By integrating the changes in various modes of transportation after scaling them according to the original values, the influence of different variables on the final output variable can be assessed. As some of the input variables, including HS 2-digit, HS 4-digit, common official language, land contiguous,

landlock, and FTA, are qualitative variables, a 10% increase or decrease in their values has no significance. Therefore, the sensitivity of these variables is not considered.

Table S9 displays the analysis results for the selected 12 variables of the "No Land" dataset and 16 variables of the "Land" dataset. For instance, a 10% of variation in the importer's maritime infrastructure quality in the "No Land" dataset will result in an average 10.8% change in the output. In both datasets, the transportation infrastructure quality of the exporter and importer is the most significant factor influencing the final output, while the trade value and volume of each flow have minimal impacts, especially in "Land" dataset. This may be due to the relatively small initial values of trade value and volume at the input level, resulting in insignificant changes after modification. Infrastructure quality will determine whether the commodity is suitable for transportation from decision-making and economic feasibility perspectives, thus producing the highest impact. In terms of infrastructure quality, maritime and air transportation have the greatest influence, indicating that the quality of ports and airports significantly affects mode choice of freight transportation in the ANN.

The aviation CO_2 emissions intensity

The International Council of Clean Transportation (ICCT) calculated the passenger CO_2 intensity by region route group for the year 2019, and differences could be noticed between regions. To incorporate the regional heterogeneity in carbon intensity, we decide to calculate region route-based freight carbon intensity.

According to the report, the average CO_2 intensity of passenger is 90 gCO_2/RPK. The percentage difference between every region route group and the average value was then calculated using the following equation:

$$diff_{rg} = \frac{absolute\ value_{rg} - 90}{90} \times 100\%$$

We also calculate the average CO_2 intensity of freight based on the total transportation work (tonne-km) and total emissions provided in the report:

$$\text{Average freight CO}_2\text{ intensity} = \frac{135 \times 10^6\ tonnes\ CO_2}{253 \times 10^9\ tonne \cdot km} \times 10^6 = 533.6\ gCO_2/tonne \cdot km$$

To obtained the specific freight CO_2 intensity of each region route group, we assume that the difference in passenger and freight load factor between region routes is consistent. Thus, the freight CO_2 emission intensity can then be achieved by the following formula:

$$\text{Freight CO}_2\text{intensity of route} = 533.6 + (533.6 \times diff_{rg})$$

The calculation results are listed in **table S10**.

International voyage identification and emission calculation

The identification of international voyages is based on the vessel speed and navigation trajectory recorded by AIS signals. To align with the ship types in maritime trade, the activity data of non-cargo vessels in the AIS data is first removed, and some ship types are merged. Finally, nine types of transport vessels are used, including container, bulk, oil tankers, miscellaneous, chemical tanker, liquefied gas tanker, RORO, general cargo, and reefer.

The identification process of maritime voyages is as follows: predefined port boundaries are established for each country or region. For the annual AIS trajectory of each vessel, the instantaneous speed and relative position of every AIS signal point with respect to the port boundaries are examined. The continuous AIS trajectory is then segmented into voyages

between departure and arrival countries. A continuous AIS trajectory is defined as one voyage if it meets the following criteria: a) the first and last AIS signals of the trajectory are located within the port boundaries of two different countries; b) the vessel speeds at both the departure and arrival AIS signals are less than 1 knot, indicating berthing status; and c) all AIS signals during navigation between the two countries show speeds greater than 1 knot.

Here, the definition of port boundaries is critical to the accuracy of the results. Major maritime trading countries (e.g., China, the United States) possess multiple non-contiguous coastlines with hundreds of coastal ports, and each port may contain several berths. As this study focuses on international trade, we did not delineate buffers for individual berths; instead, all berthing areas of ports located along the same national coastline were aggregated into a single boundary. For island nations (e.g., Indonesia), separate delineations were applied to each island group. Considering the positional uncertainties of AIS signals, the delineation of coastal areas followed four principles: (1) covering the major berthing and anchorage areas of the majority of ports; (2) excluding nearby shipping lanes; (3) avoiding overlap or conflicts with neighboring countries; and (4) when the above requirements were satisfied, extending the boundary to the national exclusive economic zone.

The shipping CO_2 emissions for voyage i, classified by vessel type v and size bin b was calculated based on the team's previously developed Shipping Emission Inventory Model (SEIM v2.0). SEIM is a disaggregate dynamic method driven by drive the high-frequency ship AIS data and the integrated Ship Technical Specifications Database (updated to 2020). The emission calculation in this study was made for each individual vessel, with a breakdown into three different engine types (main engine, auxiliary engine, and boiler) and four operation modes (at berth, at anchorage, maneuvering, and at sea). The equations applied for emission calculations follow those described in our previous studies (*54*).

<u>CO_2 emissions validation</u>

Although international trade-linked transportation emissions have gained increasing attention, there is not much research focusing on a similar scope for comparison. Currently, the known data for international trade transportation emissions mainly pertains to maritime and aviation transportation. **Fig. S9** provides the comparison results of shipping and aviation emissions estimates in this study with those from other studies. For shipping emissions, **Fig. S9A** reveals that, for the years after 2019, the results of this study show good correlation with those calculated by the IEA. Additionally, the emissions calculated by this study for 2019 are comparable to those calculated for 2015 by another study (*21*). For the period before 2009, the results of this study are significantly lower than those of other studies, due to three main reasons. First, differences in calculation methods. For example, IEA estimated emissions based on fuels data reported by countries and industries, while the IMO Third GHG Study adopted both bottom-up and top-down methods. The top-down estimate primarily used data on marine bunker sales sourced from IEA, and the bottom-up estimate combined global fleet technical data with fleet activity data derived from AIS observations. Second, the estimated transportation modal share data used in this study was an average data of recent years, which might differ from the actual modal share pattern in years 1995, 2000, 2008, and 2009. Third, there were varying maritime trade volume and turnover records between different studies. As IEA and IMO didn't provide their corresponding data, this study referred to data from the BACI. **Fig. S10** further demonstrates that shipping trade volume and turnover data in this study are lower than those reported by other sources. Specifically, the trade volume figures from this study fell below the figures reported by United Nations Conference on Trade and Development (UNCTAD) across the examined years. This trend was mirrored in the trade turnover data, where this study's estimates were also lower when compared to UNCTAD (*55*), Wang et al.

(*21*), and VoySEIM. Several factors could explain these discrepancies, including differing methodologies, the scope of data collection, and the types of shipping activities considered.

For aviation, **Fig. S10B** shows that the results of this study for the year 2019 are higher than those calculated by the ICCT. ICCT used the Global Aviation Carbon Assessment (GACA) model, which merged multiple publicly available data, including airline operations, airports, and capacity, to estimate the fuel consumption and CO_2 emissions (*35*). Additionally, for the years 2015 and 2004, which are similar to the period studied, the results of this study are also relatively higher. However, overall, the differences with other studies are less than 30%, which is within an acceptable range.

Additionally, we compared the total trade-linked transportation emissions in 2019 for ten countries, as calculated in this study, with results in 2015 study (*21*). As shown in **Fig. S10C**, the two studies exhibit good correlation, with an R^2 exceeding 0.9, further demonstrating the reliability of the country-scale results of this study.

<u>The interannual variation of transportation modes and CO_2 emissions of global trade</u>

Our model traces back to 1995, coinciding with the acceleration of global trade. Since 1995, the world has witnessed substantial economic expansion, with significant growth in trade value, trade transportation and global GDP. However, the improved fuel efficiency has mitigated the impact of increased trade volume, keeping the international trade-linked transportation CO_2 emissions have relatively stable during this period. Specifically, they increased 15.4% from 1995 to 2008 and experienced a brief drop of 11.7% in the 2008-2009 period impacted by global economic recession. The international trade-linked transportation CO_2 emissions in 2019 was 1004 Mt, which is comparable with the 1140 Mt in 2015 reported by Wang et al (*21*). Between 2019 and 2021, economic stagnation due to the COVID-19 pandemic has impacted the global trade and related emissions with a 4.7% decline in 2020, followed by a slight rebound to 971 Mt as trade recovered in 2021. Additionally, the share of international trade-linked transportation CO_2 emissions in global transportation emissions overall showed a decreasing trend from 1995 (19.2%) to 2021 (12.6%), indicating the shift in international transportation to lower emission intensity modes. The temporary increase in this share in 2020 was primarily due to the greater stability of international trade-linked transportation compared to domestic transportation under the influence of COVID-19.

Fig. 2B shows the evolution of trade-linked CO_2 emissions from different transportation modes over the past 26 years. In 1995, shipping accounted for only 30% of emissions, while aviation contributed the most with 56%. The contributions of shipping and aviation to emissions have shown a reciprocal trend, with the share of shipping increasing to 65% in 2019 and the share of aviation decreasing to 21%. This change is mainly due to the nearly 200% increase in the transportation work (as defined in the **Methods. International trade-linked transportation emissions calculation**) of shipping during this period, while the transportation work of aviation decreased by nearly half (**Fig. S3**). The shares of road and rail emissions remained stable during this period at 13% and 1%, respectively. Under the impact of COVID-19, the emissions from different transportation modes decreased to varying degrees, with shipping, aviation, road, and rail decreasing by 3%, 9%, 1%, and 21%, respectively from 2019 to 2020. Although road transportation overall was less affected globally, the emissions from road transportation decreased most significantly in East Asia by 32%, due to stricter lockdown policies to prevent the spread of the pandemic (*56*). However, it is observed that operations of transportation modes, except aviation, quickly recovered from the impact of COVID-19. Further decomposing the aviation transportation work of different HS two-digit commodities helps identify those leading this change, such as iron and steel (HS 72), electrical machinery

and equipment (HS 85), machinery and mechanical appliances (HS 84), organic chemicals (HS 29), and food industries (HS 23) (**Fig. S11**).

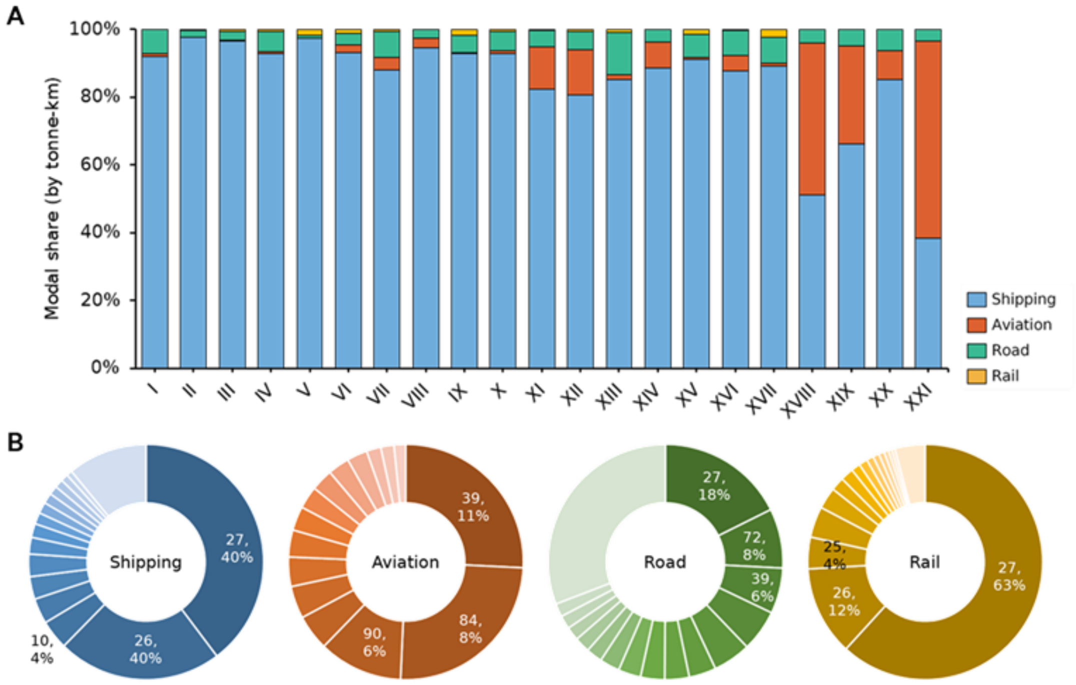

Fig. S1.

Transportation modal share profile in global trade in the commodity level. (**A**) Grouped by commodity; (**B**) grouped by transportation modes.

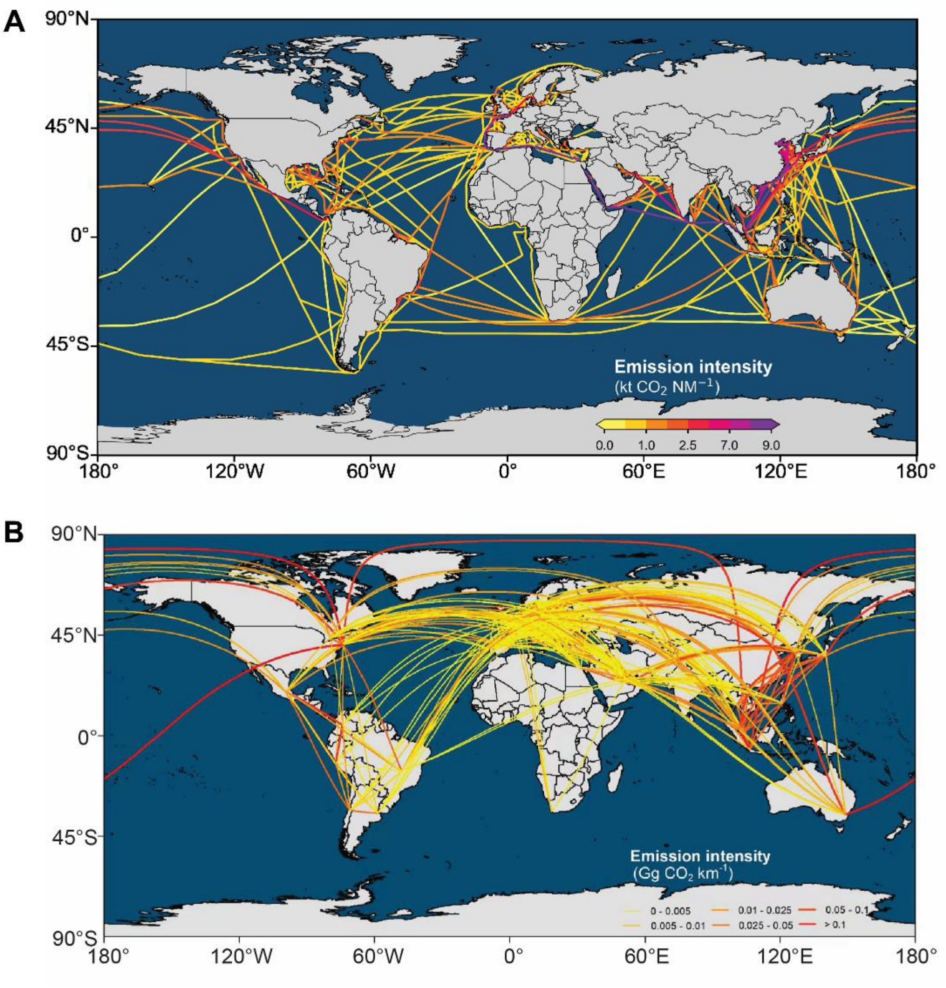

Fig. S2.

Global maps of international trade-linked emissions of shipping and aviation. For (**A**), the shipping route network is established based on the shortest paths of the world's major ports. The emissions intensity ($GgCO_2NM^{-1}$) represents the cumulative emissions of all trade transport vessels passing through each route segment. For (**B**), the aviation route network is constructed based on the busiest routes between countries.

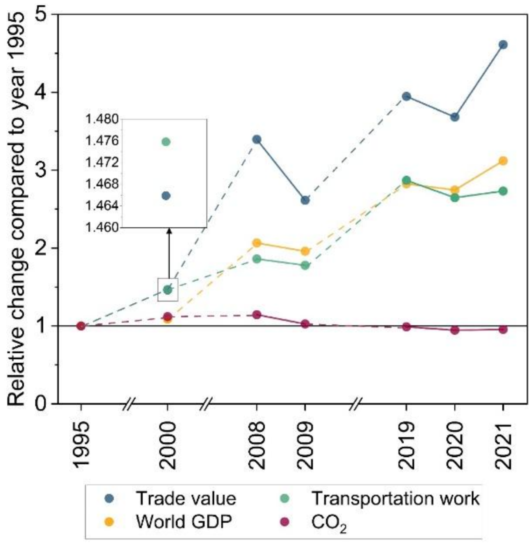

Fig. S3.

The world GDP, trade value, transportation work, and international trade-linked transportation CO_2 emissions. The baseline is established at the value for the year 1995 (= 1).

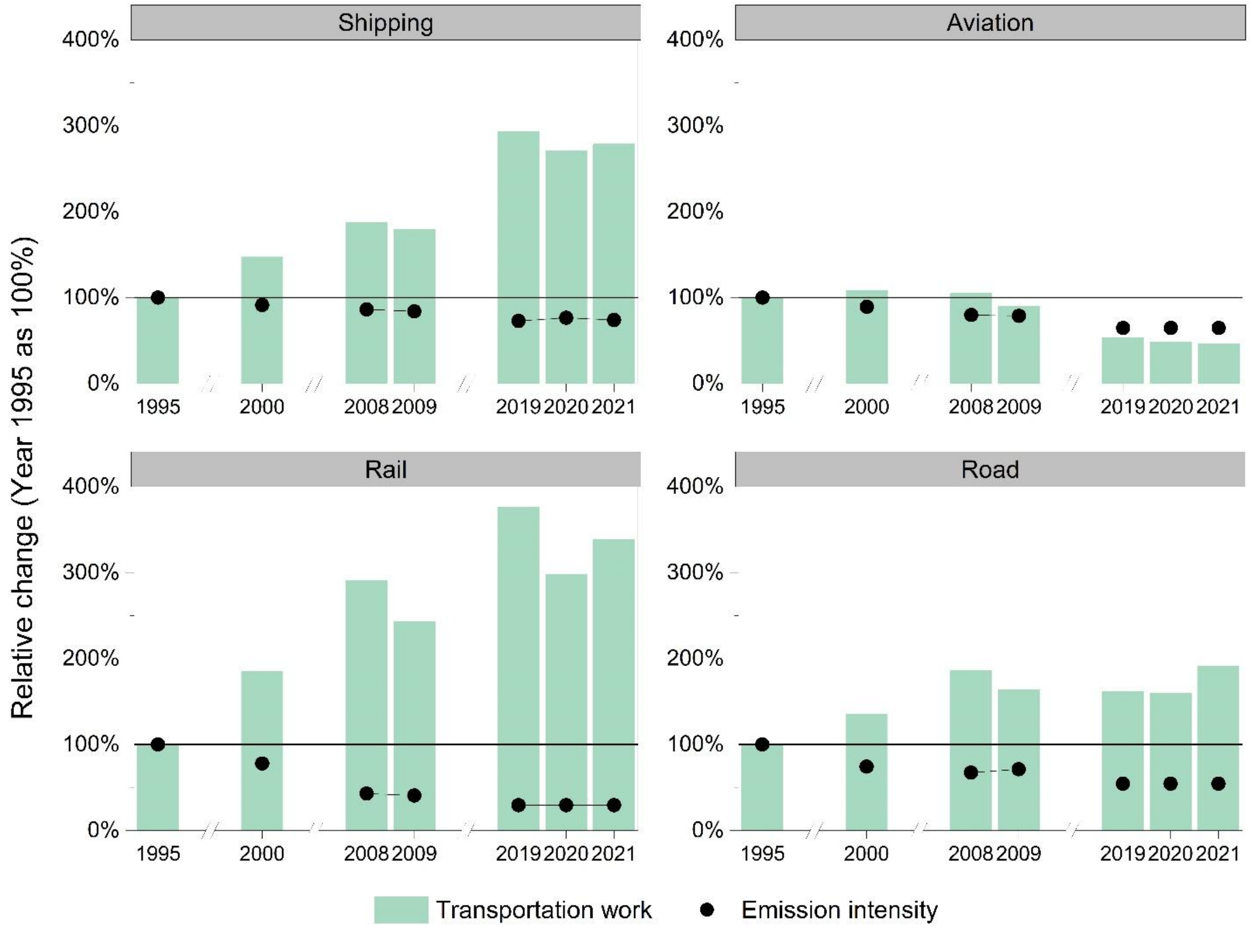

Fig. S4.

Relative change of transportation work and emission intensity of shipping, aviation, rail, and road. The value of the year 1995 is set as 100%. The emission intensity in this figure represents a global average value.

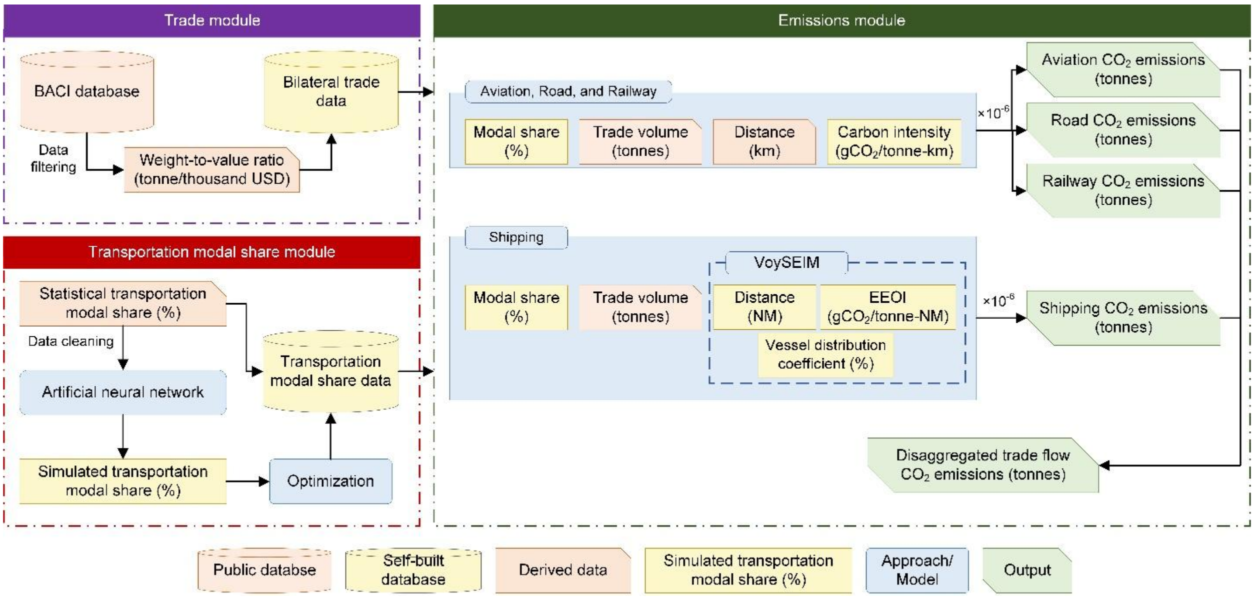

Fig. S5.

The model structure.

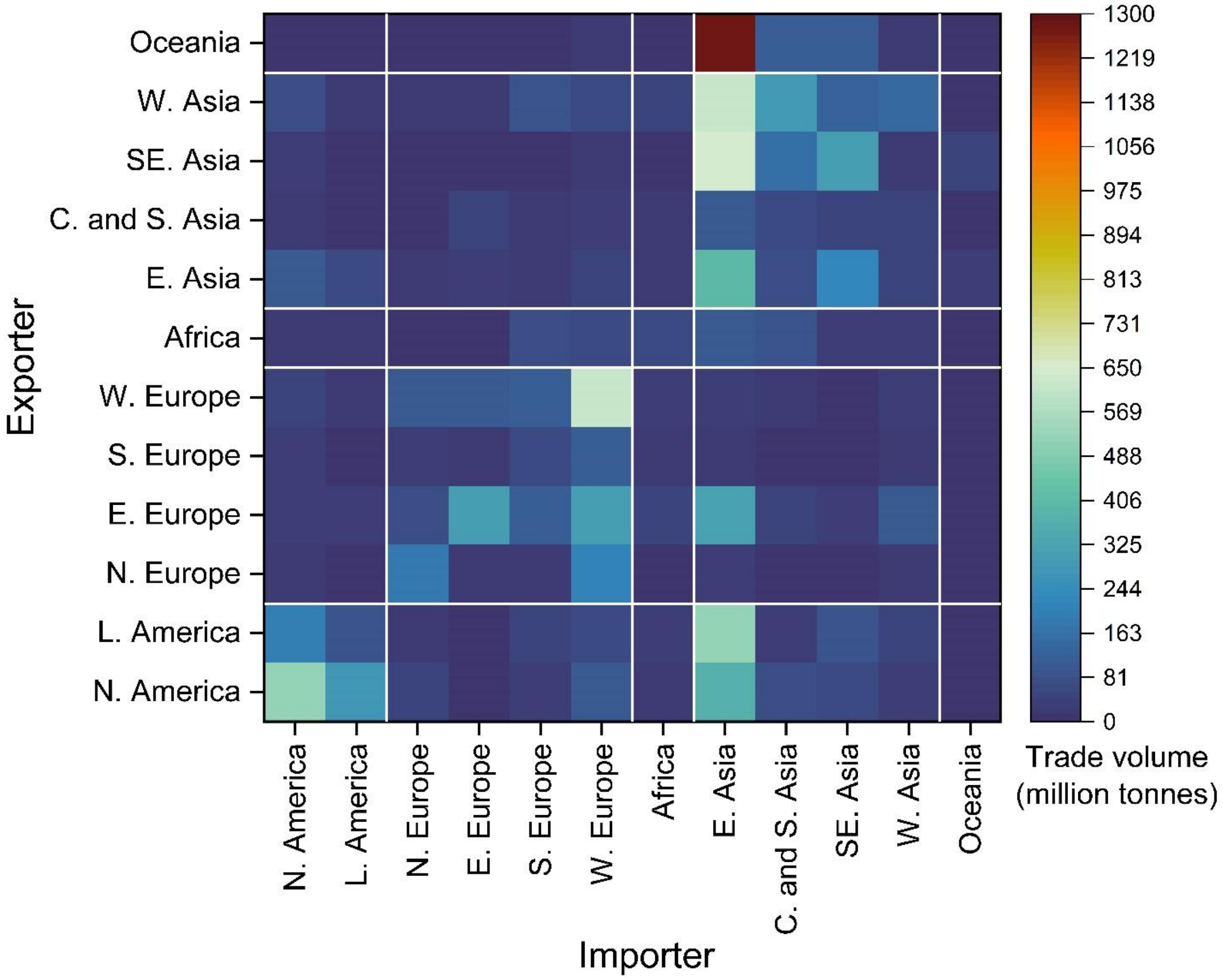

Fig. S6.

The structure of trade volume data in the model, aggregated at regional level. The region grouping is provided in Table S2.

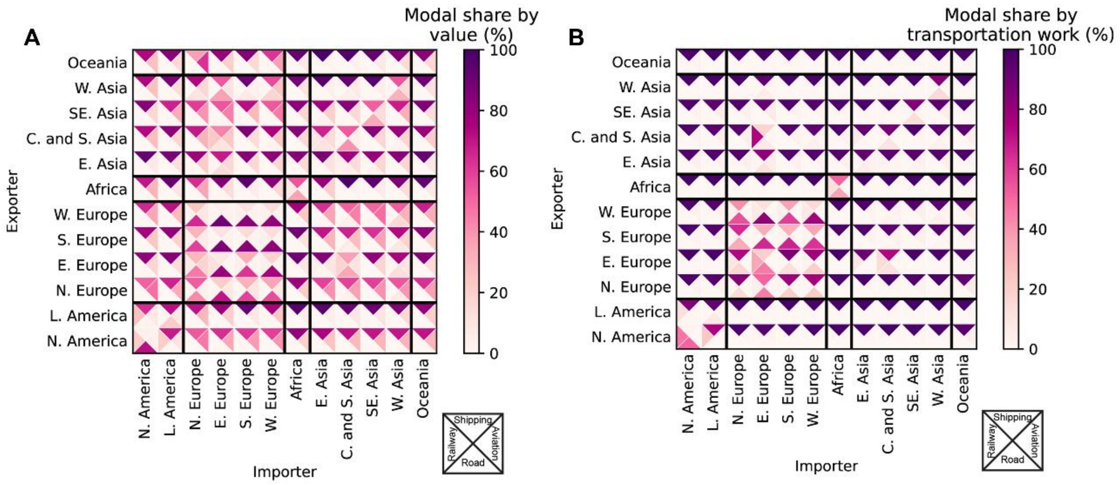

Fig. S7.

The structure of transportation modal share data in the model, aggregated at regional level. The region grouping is provided in Table S2.

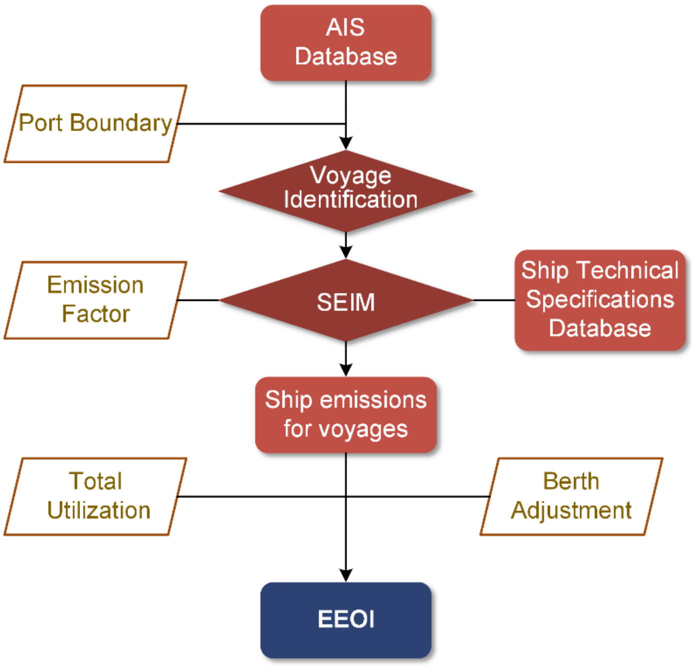

Fig. S8.
Flowchart of EEOI calculation

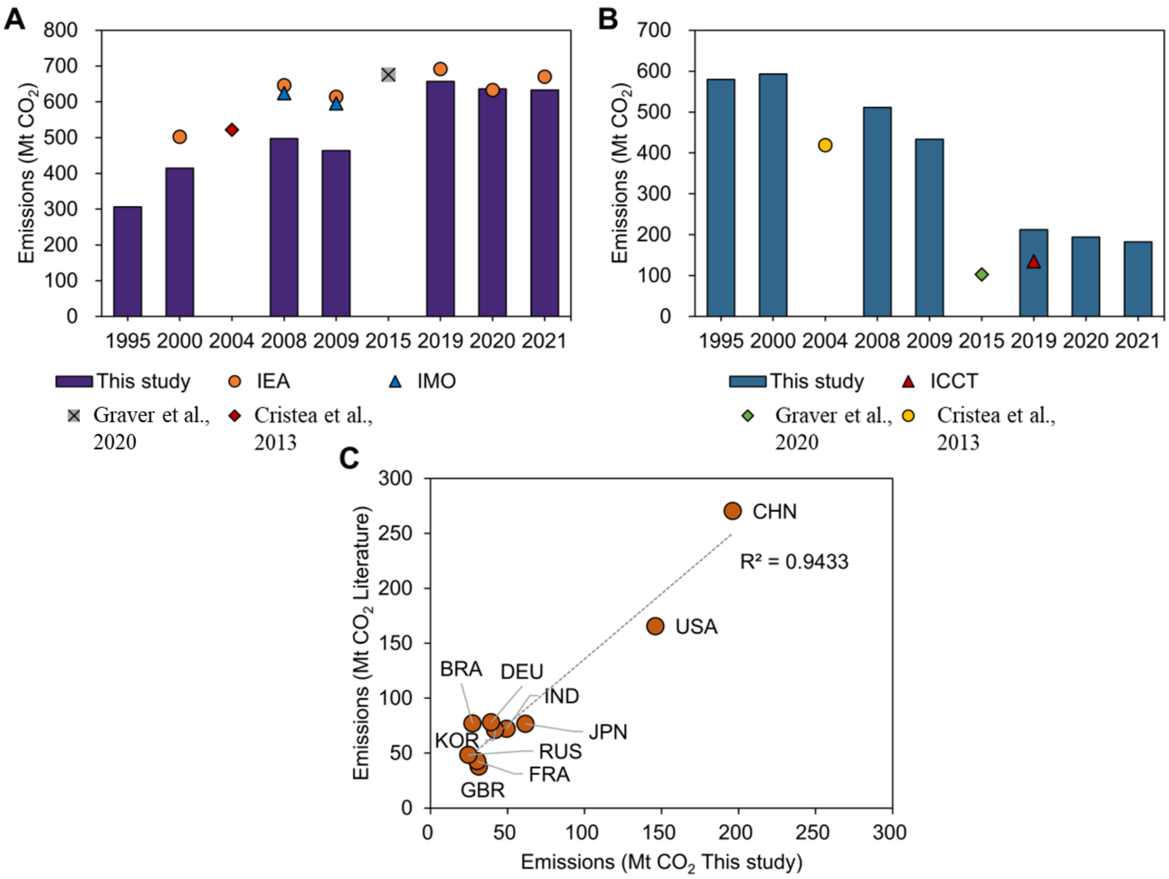

Fig. S9.

Emissions validation. Comparison of international (**A**) shipping and (**B**) aviation emissions results between this study and other studies, and (**C**) comparison of emissions for ten countries between this study and other study.

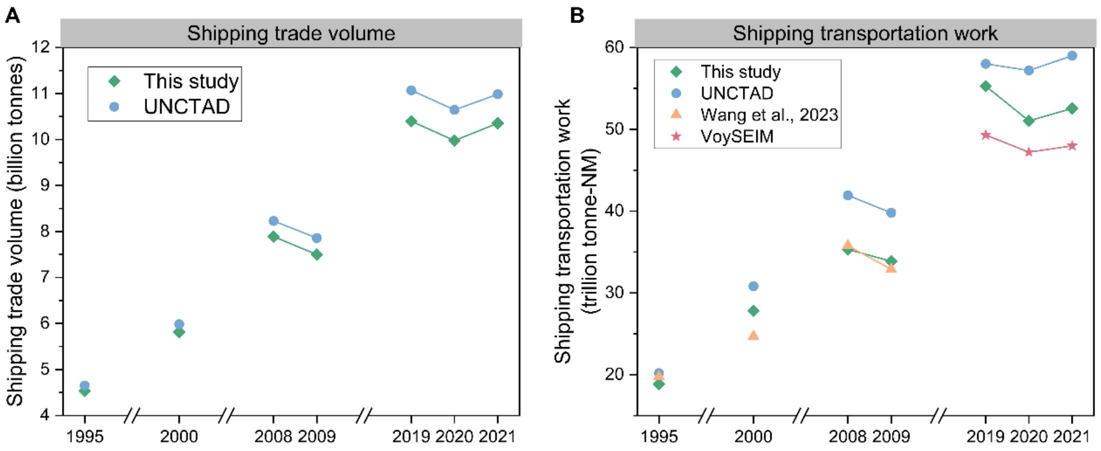

Fig. S10.

Comparison of (A) shipping trade volume and (B) transportation work. We compare our data with the Review of Maritime Transport by UNCTAD (*56*), Wang et al., 2023 (*36*), and VoySEIM.

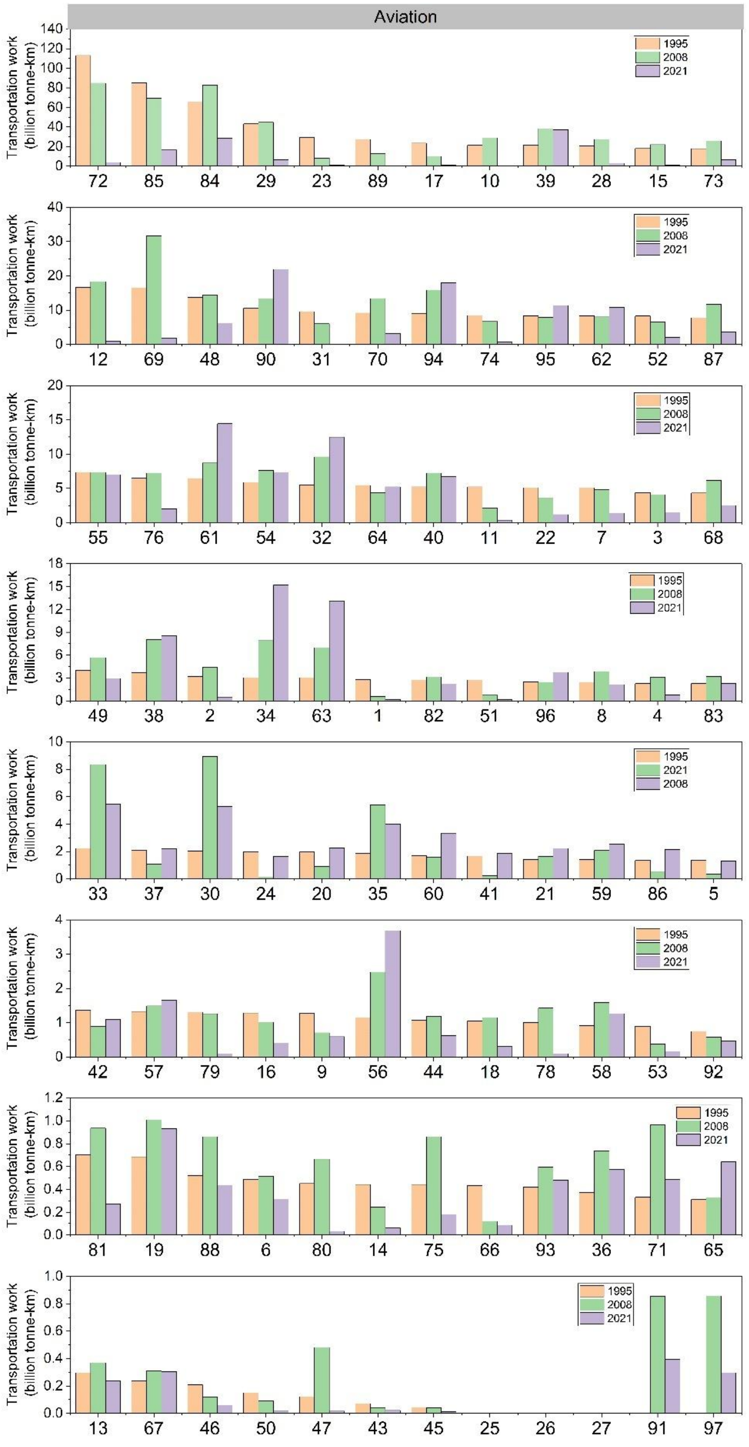

Fig. S11.

The aviation transportation work of HS two-digit-level commodities in years 1995, 2008, and 2021. The commodities (in HS two-digit level) are arranged in descending order of aviation transportation work in the year 1995.

Table S1.

HS four-digital code of top 144 traded goods sorted by weight in the CHN (exporter) -CZE (importer) trade pair.

1207	6815	2008	3005	2921	2918	4421	2903	7210	6910	6202	7020
8480	7615	8506	7315	9603	3921	2811	7019	2106	6912	6115	6302
3604	3002	8207	7604	8429	6210	6911	6110	9404	3907	8422	7606
3920	8427	6203	6116	6802	2309	2922	8407	7314	7013	2606	8534
8205	8505	4819	7307	8701	8432	6306	9406	8482	8104	8425	6810
4810	8466	8716	9019	6403	4016	3924	8711	6902	805	9504	8474
8443	7321	7610	6404	7228	8703	7229	6402	2920	8545	7616	2519
3506	8424	8465	8421	7323	8479	7325	8538	5603	8413	8477	7010
8483	4202	8508	8509	8536	7308	3824	5402	3923	8450	8714	7607
8467	4011	8541	9405	7007	8529	7217	8433	8481	8518	8415	6307
8418	8431	3926	3918	8544	8504	8503	8501	8517	8414	8302	8507
2201	8708	9506	8528	7318	9401	9403	8471	8516	9503	7326	8473

Notes

The numbers in Table S1 represent the HS four-digit codes, and their positions correspond to the arrangement of the matrix in Figure 2c. For example, the HS code 2201 in the bottom-left corner of Table S1 represents the commodity with the highest weight, corresponding to the value in the bottom-left corner of the matrix in Figure 2c.

Table S2.

GDP, trade value, trade volume ranking of selected economies and region grouping.

No.	ISO Alpha-3 code	Economies	2019			2020			2021			Region group[3]
			GDP ranking[1]	Trade value ranking[2]	Trade volume ranking[2]	GDP ranking[1]	Trade value ranking[2]	Trade volume ranking[2]	GDP ranking[1]	Trade value ranking[2]	Trade volume ranking[2]	
1	DZA	Algeria	56	62	46	57	66	52	58	66	48	Africa
2	ARG	Argentina	27	46	37	30	47	39	28	49	37	Latin America
3	AUS	Australia	13	23	3	13	22	3	13	22	3	Oceania
4	AUT	Austria	28	31	41	26	29	41	30	31	40	Western Europe
5	BGD	Bangladesh	41	49	48	31	51	47	33	50	43	Central and South Asia
6	BLR	Belarus	77	63	44	77	64	46	74	64	58	Eastern Europe
7	BEL	Belgium	24	13	15	24	12	18	24	13	16	Western Europe
8	BRA	Brazil	9	26	8	12	27	6	12	26	6	Latin America
9	CAN	Canada	10	10	11	9	10	10	9	11	11	North America
10	CHL	Chile	42	43	47	45	43	45	44	42	46	Latin America
11	CHN	China	2	1	1	2	1	1	2	1	1	East Asia
12	COL	Colombia	39	55	34	44	59	37	45	56	42	Latin America
13	CZE	Czechia	47	28	40	47	28	42	48	29	38	Eastern Europe
14	DNK	Denmark	38	36	58	35	35	56	36	38	54	Northern Europe
15	EGY	Egypt	40	53	45	32	50	44	35	48	44	Africa
16	FIN	Finland	44	42	50	43	42	55	46	44	55	Northern Europe
17	FRA	France	7	5	13	7	5	14	7	6	14	Western Europe
18	DEU	Germany	4	3	7	4	3	7	4	3	7	Western Europe
19	GRC	Greece	53	51	56	52	48	53	52	41	36	Southern Europe
20	HKG	Hong Kong SAR, China	35	14	42	39	13	38	40	15	51	East Asia
21	HUN	Hungary	56	34	55	55	34	54	56	34	56	Eastern Europe

No.	ISO Alpha-3 code	Economies	2019			2020			2021			Region group[3]
			GDP ranking[1]	Trade value ranking[2]	Trade volume ranking[2]	GDP ranking[1]	Trade value ranking[2]	Trade volume ranking[2]	GDP ranking[1]	Trade value ranking[2]	Trade volume ranking[2]	
22	IND	India	5	12	5	6	14	5	5	10	5	Central and South Asia
23	IDN	Indonesia	16	30	9	16	30	9	16	28	10	Southeast Asia
24	IRN	Iran, Islamic Rep.	45	66	53	48	72	72	42	78	75	Central and South Asia
25	IRQ	Iraq	50	44	30	53	46	31	53	47	31	West Asia
26	IRL	Ireland	30	33	65	28	33	65	26	33	67	Northern Europe
27	ISR	Israel	31	45	70	29	44	66	27	45	65	West Asia
28	ITA	Italy	8	9	16	8	9	16	8	8	17	Southern Europe
29	JPN	Japan	3	4	6	3	4	8	3	4	8	East Asia
30	KAZ	Kazakhstan	53	47	33	54	49	32	55	57	41	Central and South Asia
31	KWT	Kuwait	58	54	36	63	60	40	60	61	39	West Asia
32	MAC	Macao SAR, China	83	123	49	103	134	49	100	127	52	East Asia
33	MYS	Malaysia	34	24	18	41	25	17	39	24	18	Southeast Asia
34	MEX	Mexico	15	11	21	15	11	20	15	12	21	Latin America
35	MAR	Morocco	60	57	51	59	54	48	59	58	53	Africa
36	MOZ	Mozambique	123	100	75	127	104	59	125	93	50	Africa
37	NLD	Netherlands	17	7	12	17	6	11	17	5	9	Western Europe
38	NZL	New Zealand	51	58	67	50	58	68	50	53	64	Oceania
39	NGA	Nigeria	26	50	39	27	55	43	31	55	45	Africa
40	NOR	Norway	30	37	28	33	38	28	29	35	29	Northern Europe
41	OMN	Oman	72	64	43	71	61	36	68	60	34	West Asia
42	OAS	Other Asia[4]	21	17	25	21	15	25	21	14	25	East Asia

No.	ISO Alpha-3 code	Economies	2019			2020			2021			Region group[3]
			GDP ranking[1]	Trade value ranking[2]	Trade volume ranking[2]	GDP ranking[1]	Trade value ranking[2]	Trade volume ranking[2]	GDP ranking[1]	Trade value ranking[2]	Trade volume ranking[2]	
43	PAK	Pakistan	43	60	54	42	57	50	43	52	47	Central and South Asia
44	PER	Peru	50	56	59	51	56	62	51	54	62	Latin America
45	PHL	Philippines	33	35	32	34	36	33	38	36	32	Southeast Asia
46	POL	Poland	22	21	29	22	20	29	22	20	28	East Europe
47	PRT	Portugal	49	41	57	49	41	57	49	43	57	Southern Europe
48	QAT	Qatar	55	52	38	58	52	35	57	51	33	West Asia
49	KOR	Republic of Korea	12	8	10	10	8	12	10	7	12	East Asia
50	ROU	Romania	48	39	52	46	39	51	47	40	49	Eastern Europe
51	RUS	Russian Federation	11	16	4	11	19	4	11	17	4	Eastern Europe
52	SAU	Saudi Arabia	18	29	14	20	32	13	18	30	13	West Asia
53	SGP	Singapore	34	18	22	38	21	24	37	19	23	Southeast Asia
54	SVK	Slovakia	62	40	61	62	40	61	61	39	59	Eastern Europe
55	ZAF	South Africa	37	38	23	40	37	26	32	37	27	Africa
56	ESP	Spain	14	15	20	14	16	21	14	16	19	Southern Europe
57	SWE	Sweden	25	32	35	23	31	34	23	32	35	Northern Europe
58	CHE	Switzerland	20	19	60	18	17	58	20	21	60	Western Europe
59	THA	Thailand	23	25	27	25	23	27	25	25	26	Southeast Asia
60	TUR	Turkey	19	27	24	19	26	22	19	27	22	West Asia
61	UKR	Ukraine	58	48	31	56	45	30	54	46	30	Eastern Europe
62	ARE	United Arab Emirates	29	22	17	36	24	15	34	23	15	West Asia
63	GBR	United Kingdom	6	6	19	5	7	19	6	9	20	Northern Europe
64	USA	United States	1	2	2	1	2	2	1	2	2	North America

65	VNM	Vietnam	45	20	26	37	18	23	41	18	24	Southeast Asia

Notes:

1. GDP data is from The World Bank (https://data.worldbank.org/indicator/NY.GDP.MKTP.CD).

2. Trade value and volume data are from the Base pour l'Analyse du Commerce International (BACI) database (http://www.cepii.fr/CEPII/en/bdd_modele/bdd_modele_item.asp?id=37)

3. Region group is categorized according to the geographic regions defined by the United Nations Statistics Division: https://unstats.un.org/unsd/methodology/m49/.

4. GDP, trade value, and trade volume of Other Asia is a proxy of Taiwan, Chin

Table S3.
The emissions intensity of transportation modes in different years.

Mode	Year	Source	CO_2 emissions intensity
Aviation[1]	1995	Author's calculation based on the IATA's tracking on aviation efficiency performance: 54.3% of improvement between 1990 and 2019.	823.3 g/tonne-km
	2000	Author's calculation based on the IATA's tracking on aviation efficiency performance: 37.8% of improvement between 2000 and 2019.	735.3 g/tonne-km
	2008	Author's interpolation based on estimated values from the years 2000 and 2009.	657.5 g/tonne-km
	2009	Author's calculation based on the IATA's tracking on aviation efficiency performance: 21.4% of improvement between 2009 and 2019.	647.8 g/tonne-km
	2019-2021	Refer to Supplementary Method 3	533.6 g/tonne-km
Rail	1995	Whitelegg, John (1993), Transport for a Sustainable Future -- The case for Europe	48.0 g/tonne-km
	2000	Author's interpolation based on estimated values from the years 1995 and 2008.	37.4 g/tonne-km
	2008	https://www.eea.europa.eu/data-and-maps/daviz/specific-co2-emissions-per-tonne-2#tab-chart_1	20.6 g/tonne-km
	2009		19.5 g/tonne-km
	2019-2021	https://www.cbo.gov/publication/58861	14.1 g/tonne-km
Road	1995	Whitelegg, John (1993), Transport for a Sustainable Future -- The case for Europe	207.0 g/tonne-km
	2000	https://www.eea.europa.eu/data-and-maps/daviz/specific-co2-emissions-per-tonne-2#tab-chart_1	153.7 g/tonne-km
	2008	https://www.eea.europa.eu/data-and-maps/daviz/specific-co2-emissions-per-tonne-2#tab-chart_1	139.0 g/tonne-km
	2009	https://www.eea.europa.eu/data-and-maps/daviz/specific-co2-emissions-per-tonne-2#tab-chart_1	147.4 g/tonne-km
	2019-2021	https://www.cbo.gov/publication/58861	112.8 g/tonne-km
Shipping[2]	1995	Author's estimation based on IMO Fourth GHG Study 2020	16.2 g/tonne-NM
	2000		14.9 g/tonne-NM
	2008		14.1 g/tonne-NM

2009		13.7 g/tonne-NM
2019	VoySEIM's results	11.9 g/tonne-NM
2020	VoySEIM's results	12.5 g/tonne-NM
2021	VoySEIM's results	12.0 g/tonne-NM

Notes:

1. The emission intensity of aviation given in this table is a global average value. A region-specific route value is used when calculating emissions.
2. The emission intensity of shipping given in this table is a global average value. In calculating emissions from 1995 to 2009, we utilize emission intensity that distinguishes between different vessel types. While for the years 2019 to 2021, VoySEIM provides emission intensity that differentiates between exporter-importer-vessel types.

Table S4.

Trade module details.

Year	Number of HS4 commodity	Number of economies	Share of selected economies (%)			Number of trade flow
			GDP	Trade value	Trade volume	
1995			96	84	84	1127059
2000			96	91	85	1567504
2008			95	89	86	1859028
2009	1221	65	95	89	86	1820880
2019			96	94	92	1997133
2020			96	95	92	1983752
2021			96	94	92	2047466

Table S5.

The training hyperparameters of the ANN.

Training hyperparameters	"No Land" Dataset	"Land" Dataset
Normalization method	Min-max method	
Learning rate	0.01	
Training function	Levenberg-Marquardt backpropagation	
Maximum epoch	1000	
Maximum validation check	6	
Number of input layers	19	23
Number of hidden layers	4	4
Number of neuron nodes in hidden layer	15, 15, 13, 10	18, 15, 13, 10
Activation function of hidden layer	ReLU	
Number of output layers	2	4
Activation function of output layer	tansig	

Table S6.

The prediction performance of the ANN.

Year	Dataset	MSE	R	CA
2019	No Land	0.12307	0.63806	77.3%
	Land	0.048351	0.83945	82.6%
2020	No Land	0.12831	0.6215	75.7%
	Land	0.047569	0.8402	83.1%
2021	No Land	0.12437	0.64303	75.9%
	Land	0.048772	0.83077	81.3%

Table S7.

Comparison of regional modal share (%).

Region	By value				By kg–km			Year 2004
Modal shares by exporter	Shipping	Aviation	Rail	Road	Shipping	Aviation	Rail	Road
North America	63.9(46.6)	15.1(21)	8.0(6.7)	12.9(25.7)	92.2(91.8)	1.5(1.4)	4(1.4)	2.3(5.5)
South America	67.7(66.4)	30.8(22.7)	0.8(0.3)	1.4(10.6)	84.7(96.0)	14.1(1.3)	0.1(0.2)	1.1(2.6)
Europe	35.8(35.5)	10.6(13)	2.4(4.5)	51.2(46.9)	83.8(91.7)	1.1(1.1)	4.3(2.0)	10.8(5.2)
South Asia	76.3(74.8)	15.9(21.7)	2.1(0.6)	5.7(2.9)	97.2(99.2)	0.4(0.5)	1.1(0.1)	1.2(0.3)
East Asia	82.7(72.8)	15.5(25.8)	1.0(0.2)	0.8(1.2)	99.2(98.8)	0.2(1.1)	0.5(0)	0.1(0.1)
Middle East/Africa	83.2(68.2)	15.4(19.1)	0.2(0)	1.2(12.7)	95.7(88.5)	1.3(0.9)	0.6(0)	2.2(10.6)
Oceania	93.0(78.0)	17.0(22)	0(0)	0(0)	97.8(98.1)	2.2(1.9)	0(0)	0(0)
Modal shares by exporter								
North America	50.7(28.3)	20.6(25.9)	9.4(9.4)	19.3(36.4)	94.0(88.2)	0.8(4.6)	4.0(1.4)	1.3(5.8)
South America	82.7(85.7)	15.8(7.3)	0.1(0.2)	1.4(6.8)	91.8(99.1)	6.8(0.2)	0.2(0)	1.2(0.6)
Europe	35.7(35.1)	12.3(13.0)	2.5(4.5)	49.5(47.3)	83.8(89.3)	1.2(0.9)	2.4(2.8)	12.6(7.1)
South Asia	72.8(73.9)	19.6(21.6)	2.9(0.8)	4.7(3.7)	86.0(97.8)	1.5(1.0)	103.(0.2)	2.2(1.0)
East Asia	84.8(72.0)	13.0(26.8)	0.7(0.2)	1.5(1.0)	95.5(98.8)	3.0(0.9)	0.2(0)	1.2(0.2)
Middle East/Africa	83.1(80.6)	15.1(9.3)	0.3(0)	1.5(10.1)	98.6(97.3)	0.1(0.1)	0.3(0)	1.1(2.6)
Oceania	92.0(89.8)	8.0(10.2)	0(0)	0(0)	99.9(99.9)	0.1(0.1)	0(0)	0(0)

Notes:

1. The numbers in the table are presented as A(B), where A represents the results (average from 2019-2021) calculated in this study, and B represents the results for 2004 from Cristea et al.'s study (*20*).

Table S8.

Comparison of national modal share by value (%).

Country	Modal shares by exporter			Modal shares by importer		
	Shipping	Aviation	Land	Shipping	Aviation	Land
ARE	68.1(69.2)	27.6(19.9)	4.3(10.9)	65.6(56.1)	32.3(35.7)	2.1(8.2)
ARG	89.6(72.6)	8.4(7.5)	2(19.9)	67.8(63.8)	30.8(19)	1.4(17.2)
AUS	73.9(86.8)	25.7(13.2)	0.4(0)	79.8(77.6)	20.2(22.4)	0(0)
AUT	42(26.6)	21.8(11.2)	36.3(62.2)	42.6(21.4)	23.9(8.9)	33.5(69.8)
BEL	48.2(29.1)	25.3(12.3)	26.4(58.6)	59(33.6)	18.6(14.2)	22.4(52.1)
BGD	44.8(58.7)	51.2(30.7)	4(10.6)	78.3(70.6)	18.2(12.2)	3.6(17.2)
BLR	59.2(29.4)	12.7(2)	28.1(68.6)	31.3(23.7)	33.9(5.6)	34.8(70.7)
BRA	89.3(84)	9(6.9)	1.7(9.1)	80.4(77.4)	18.4(15.2)	1.2(7.4)
CAN	59.8(36.6)	37.1(9.3)	3(54.1)	62.5(32.6)	33.7(18.9)	3.8(48.5)
CHE	11.6(18.4)	54(44)	34.4(37.6)	17.2(19.4)	50.1(33.6)	32.7(47)
CHL	83.8(87)	14.2(4.8)	2(8.3)	58.6(76.7)	40(12.7)	1.4(10.5)
CHN	80.8(49)	15.1(19.6)	4.1(31.4)	79.8(58.6)	16.4(15.5)	3.8(25.9)
COL	74.6(85.8)	24.6(6.5)	0.8(7.8)	60.3(79.5)	36.8(15.6)	2.9(4.9)
CZE	38.6(26.6)	21.9(7.6)	39.5(65.8)	29.9(24)	27.7(9.9)	42.5(66.1)
DEU	46(30.5)	20.3(12.5)	33.7(57)	53.6(27.4)	15.2(12.1)	31.2(60.5)
DNK	45.9(47.9)	25.1(23.7)	29(28.4)	52.9(49.8)	21.4(17.5)	25.7(32.6)
DZA	79.9(75.3)	17.9(1.2)	2.2(23.5)	75.3(63.2)	23.4(13.7)	1.3(23.1)
EGY	78(69.8)	20(10.8)	2(19.4)	77(74)	21.1(13.5)	2(12.4)
ESP	55.8(35.7)	15.2(7.5)	29(56.8)	60.9(36.2)	12.8(7.7)	26.3(56.2)
FIN	56.6(34.6)	18.9(13.1)	24.5(52.4)	52.6(29.3)	23.2(11.6)	24.2(59.1)
FRA	43.5(30.3)	25.4(16.3)	31.1(53.4)	54.2(29)	18.5(10.7)	27.3(60.3)
GBR	50.5(43.4)	30.7(27.4)	18.8(29.2)	61.3(44.9)	21.4(18.7)	17.3(36.4)
GRC	59.4(43.5)	13.2(6.7)	27.4(49.8)	65.2(32.8)	12.2(9.2)	22.6(58)
HKG	33.9(36.8)	64.7(40.4)	1.3(22.8)	56.5(35)	41.5(30.1)	2.1(34.9)
HUN	39.6(22.2)	24.3(9.4)	36.1(68.4)	32.3(20.1)	28.9(9.5)	38.8(70.4)
IDN	79.8(87.9)	19.2(12.1)	0.9(0)	76.5(88.6)	22.4(11.4)	1.1(0)
IND	71.4(66)	24.5(21)	4.1(13.1)	77.5(63.9)	20.2(20.3)	2.3(15.8)
IRL	33.9(51.8)	41.3(48.2)	24.8(0)	49.4(70.8)	28.7(29.2)	21.8(0)
IRN	62.5(76.1)	32.5(1.8)	5.1(22.1)	52.5(55.8)	40.6(15.6)	7(28.6)
IRQ	59.1(75.6)	37.8(1.7)	3.1(22.7)	65.5(30.7)	30(17.4)	4.6(52)
ISR	49.2(44.9)	48.4(38.3)	2.4(16.8)	64.1(46.9)	34.1(27.9)	1.8(25.3)
ITA	48.8(32.3)	20.8(12.9)	30.4(54.9)	61.3(29.6)	13.9(8.9)	24.8(61.6)
JPN	72.9(77.2)	26.3(22.8)	0.8(0)	75.8(79.9)	23.7(20.1)	0.5(0)
KAZ	70(38.6)	13.8(4)	16.2(57.4)	21.9(18.5)	40.7(11.5)	37.3(70)
KOR	73.5(57.6)	25.9(20.2)	0.5(22.2)	74.7(59.6)	24.7(17.4)	0.6(23)
KWT	72.4(85.9)	24.4(1.6)	3.2(12.5)	63.2(46)	34.5(23.3)	2.4(30.8)
MAC	30.9(46.8)	67(53.2)	2.1(0)	52.8(59.8)	46.2(40.2)	1(0)
MAR	65.9(45.8)	31.2(9.1)	2.8(45)	72.3(47.4)	25.9(9.7)	1.9(42.9)
MEX	57.1(27.5)	40.7(6.7)	2.2(65.7)	72.1(47.2)	26.1(11.9)	1.8(40.9)

MOZ	81.6(79.8)	17(5.3)	1.4(15)	70.4(70.2)	26.5(7.6)	3.1(22.3)
MYS	61.3(74.6)	34.6(21.5)	4.1(3.9)	68.3(76)	27.8(19.2)	3.9(4.8)
NGA	86.4(92.6)	12.2(2.5)	1.5(4.9)	82.8(75)	16.2(19.4)	1(5.6)
NLD	48.9(29.7)	22.5(11)	28.6(59.3)	62.9(35.7)	14.3(11.3)	22.8(53)
NOR	63.2(62.9)	20.3(9.5)	16.4(27.6)	51.5(47)	26.7(19.4)	21.8(33.6)
NZL	77.7(90.9)	21.7(9.1)	0.6(0)	69.1(81.4)	30.4(18.6)	0.4(0)
OMN	77.4(87.6)	19.9(2.6)	2.7(9.9)	68.8(65.6)	28.6(15.4)	2.6(19.1)
PAK	60.8(60.8)	33.8(18.1)	5.4(21.1)	78.1(66.1)	19.1(10.7)	2.9(23.3)
PER	76.4(74.4)	22.7(17.4)	0.9(8.3)	71.2(75.5)	28(13.5)	0.8(11)
PHL	53.4(72.7)	46.1(27.3)	0.5(0)	70.3(84.1)	29.4(15.9)	0.3(0)
POL	49.4(25.7)	17.5(6.7)	33.1(67.5)	51.7(23.7)	17.5(8.8)	30.8(67.5)
PRT	55.8(33.9)	18.6(8.1)	25.7(58)	58.6(27.2)	16(6)	25.4(66.8)
QAT	82.2(90.2)	13.6(2.6)	4.2(7.3)	56.9(50.5)	38.6(30.5)	4.5(19)
ROU	48.6(22.9)	17.7(8)	33.7(69.1)	48.2(17.6)	18.9(6.6)	32.9(75.8)
RUS	77.8(44)	8.8(5.7)	13.4(50.4)	50.1(24.5)	21.9(12.2)	27.9(63.4)
SAU	87.5(85.5)	9(2.4)	3.6(12)	73.6(60.7)	23.1(19.4)	3.3(19.9)
SGP	44.2(73.8)	53.6(26.2)	2.2(0)	65(71.6)	32.5(28.4)	2.5(0)
SVK	47.2(27.6)	12.3(4.9)	40.6(67.4)	36.8(23.6)	22.8(7.7)	40.4(68.8)
SWE	49.7(48.3)	22.5(20.3)	27.7(31.4)	51.8(48.4)	23.6(17.6)	24.6(34)
THA	73.6(60.7)	24.1(21.7)	2.3(17.7)	74.1(60.6)	24.1(19.3)	1.8(20.1)
TUR	69.9(37.7)	11.5(12.7)	18.7(49.7)	66(40.2)	19.2(11.6)	14.8(48.2)
TWN	54(68.2)	45.5(31.8)	0.4(0)	73.8(74.9)	25.9(25.1)	0.3(0)
UKR	70.5(37.7)	7.6(3.6)	21.9(58.7)	48.8(25.1)	22.1(6.6)	29.1(68.3)
USA	68.5(51.5)	29.4(24.3)	2.1(24.2)	76.8(55.3)	21.1(23.5)	2.1(21.2)
VNM	61(49.4)	37(26.2)	2(24.4)	73.8(49.2)	24.1(12.8)	2.1(38)
ZAF	80.9(61)	17.4(33.5)	1.6(5.5)	79.3(78.5)	19.9(19.2)	0.8(2.3)

Notes:

1. The numbers in the table are presented as A(B), where A represents the results (average from 2019-2021) calculated in this study, and B represents the results for 2015 from Verschuur et al.'s study (*57*).

2. Land includes both rail and road transportation.

3. For Shipping, Aviation, and Land transport, the average absolute errors in modal shares by exporter are 14%, 11%, and 18%, respectively. For modal shares by importer, the average absolute errors are 15%, 10%, and 21%, respectively.

Table S9.
ANN sensitivity analysis results.

Input variable	Change in output (%)	
	Land	No Land
Exporter aviation infrastructure	30.698	6.434
Exporter maritime infrastructure	26.391	8.376
Importer maritime infrastructure	20.735	10.803
Exporter geographical group	13.942	9.007
Importer railway infrastructure	12.018	NA
Importer geographical group	11.554	3.919
Exporter railway infrastructure	9.452	NA
Importer aviation infrastructure	8.089	9.643
Bilateral distance	8.053	1.307
Importer road infrastructure	4.738	NA
Exporter road infrastructure	2.558	NA
Importer GDP	1.045	1.333
Exporter GDP	0.940	1.042
WV ratio	0.083	1.890
Trade value	0.051	0.171
Trade volume	0.006	0.015

Table S10.

Region route-based freight emissions intensity.

Region route group	Passenger emissions intensity (gCO_2/RPK)[27]	Difference ($diff_{rg}$, %)	Freight emissions intensity (gCO_2/tonne-km)
Latin America/Caribbean ↔ North America	82	-8.89	486
Europe ↔ Latin America/Caribbean	82	-8.89	486
Africa ↔ Europe	84	-6.67	498
Asia/Pacific ↔ Latin America/Caribbean	85	-5.56	504
Intra-Europe	87	-3.33	516
Asia/Pacific ↔ Middle East	87	-3.33	516
Europe ↔ Middle East	87	-3.33	516
Asia/Pacific ↔ Europe	88	-2.22	522
Africa ↔ Asia/Pacific	89	-1.11	528
Intra-Asia/Pacific	89	-1.11	528
Europe ↔ North America	89	-1.11	528
Africa ↔ Middle East	91	1.11	540
Asia/Pacific ↔ North America	93	3.33	551
Middle East ↔ North America	93	3.33	551
Latin America/Caribbean ↔ Middle East	94	4.44	557
Africa ↔ Latin America/Caribbean	96	6.67	569
Intra-North America	97	7.78	575
Africa ↔ North America	97	7.78	575
Intra-Latin America/Caribbean	98	8.89	581
Intra-Africa	118	31.11	700
Intra-Middle East	118	31.11	700

REFERENCES

1. C. Capineri, T. R. Leinbach, Freight transport, seamlessness, and competitive advantage in the global economy. *Eur. J. Transp. Infrast.* **6**, (2006).

2. O. Kherbash, M. L. Mocan, A review of logistics and transport sector as a factor of globalization. *Procedia Econ. Financ.* **27**, 42–47 (2015).

3. ITF, Key Transport Statistics 2024 (2023 data). https://www.itf-oecd.org/sites/default/files/docs/itf-key-transport-statistics-2024.pdf (2024).

4. ITF, ITF Transport Outlook 2023. https://www.itf-oecd.org/itf-transport-outlook-2023 (2023).

5. UNFCCC, Shipping Aviation and Paris. https://unfccc.int/news/shipping-aviation-and-paris (2016).

6. P. Jaramillo, S. K. Ribeiro, P. Newman, S. Dhar, O. Diemuodeke, T. Kajino, D. S. Lee, S. B. Nugroho, X. Ou, A. H. Strømman, Transport. In IPCC, 2022: Climate Change 2022: Mitigation of Climate Change. Contribution of Working Group III to the Sixth Assessment Report of the Intergovernmental Panel on Climate Change [PR Shukla, J. Skea, R. Slade, A. Al Khourdajie, R. van Diemen, D. McCollum, M. Pathak, S. Some, P. Vyas, R. Fradera, M. Belkacemi, A. Hasija, G. Lisboa, S. Luz, J. Malley,(eds.)]. *Cambridge, UK and New York, NY, USA*, (2022).

7. Uniting Aviation, States adopt a net-zero 2050 global aspirational goal for international flight operations. https://unitingaviation.com/news/environment/states-adopt-a-net-zero-2050-global-aspirational-goal-for-international-flight-operations/ (2022).

8. IMO. 2023 IMO strategy on reduction of GHG emissions from ships. https://wwwcdn.imo.org/localresources/en/OurWork/Environment/Documents/annex/MEPC%2080/Annex%2015.pdf (2023).

9. F. Afonso, M. Sohst, C. M. A. Diogo, S. S. Rodrigues, A. Ferreira, I. Ribeiro, R. Marques, F. F. C. Rego, A. Sohouli, J. Portugal-Pereira, H. Policarpo, B. Soares, B. Ferreira, E. C. Fernandes,

F. Lau, A. Suleman, Strategies towards a more sustainable aviation: A systematic review. *Prog. Aerosp. Sci.* **137**, 100878 (2023).

10. G. Bezos-O'Connor, M. Mangelsdorf, C. Nickol, H. Maliska, A. Washburn, R. Wahls, Fuel efficiencies through airframe improvements, in *3rd AIAA Atmospheric Space Environments Conference*. (2011), pp. 3530.

11. K. Cullinane, J. Yang, Evaluating the costs of decarbonizing the shipping industry: A review of the literature. *J. Mar. Sci. Eng.* **10**, 946 (2022).

12. J. Dong, J. Zeng, Y. Yang, H. Wang, A review of law and policy on decarbonization of shipping. *Front. Mar. Sci.* **9**, 1076352 (2022).

13. G. Mallouppas, E. A. Yfantis, Decarbonization in shipping industry: A review of research, technology development, and innovation proposals. *J. Mar. Sci. Eng.* **9**, 415 (2021).

14. A. de la Garza, IMO's global shipping emissions climate deal: What you need to know, Time. https://time.com/6292927/imo-global-shipping-emissions-climate-deal/ (2023).

15. Climate Action Tracker. International Aviation. https://climateactiontracker.org/sectors/aviation/ (2024).

16. S. J. Davis, N. S. Lewis, M. Shaner, S. Aggarwal, D. Arent, I. L. Azevedo, S. M. Benson, T. Bradley, J. Brouwer, Y.-M. Chiang, C. T. M. Clack, A. Cohen, S. Doig, J. Edmonds, P. Fennell, C. B. Field, B. Hannegan, B.-M. Hodge, M. I. Hoffert, E. Ingersoll, P. Jaramillo, K. S. Lackner, K. J. Mach, M. Mastrandrea, J. Ogden, P. F. Peterson, D. L. Sanchez, D. Sperling, J. Stagner, J. E. Trancik, C.-J. Yang, K. Caldeira, Net-zero emissions energy systems. *Science* **360**, eaas9793 (2018).

17. Z. Liu, J. Meng, Z. Deng, P. Lu, D. Guan, Q. Zhang, K. He, P. Gong, Embodied carbon emissions in China-US trade. *Sci. China Earth Sci.* **63**, 1577–1586 (2020).

18. W. S. van der Loeff, J. Godar, V. Prakash, A spatially explicit data-driven approach to calculating commodity-specific shipping emissions per vessel. *J. Clean. Prod.* **205**, 895–908 (2018).

19. X.-T. Wang, H. Liu, Z.-F. Lv, F.-Y. Deng, H.-L. Xu, L.-J. Qi, M.-S. Shi, J.-C. Zhao, S.-X. Zheng, H.-Y. Man, Trade-linked shipping CO_2 emissions. *Nat. Clim. Chang.* **11**, 945–951 (2021).

20. A. Cristea, D. Hummels, L. Puzzello, M. Avetisyan, Trade and the greenhouse gas emissions from international freight transport. *J. Environ. Econ. Manag.* **65**, 153–173 (2013).

21. Y. Wang, J. Liu, D. Guan, J. Meng, Z. Liu, S. Xiang, H. Yang, X. Fu, X. Hu, Q. Yang, The volume of trade-induced cross-border freight transportation has doubled and led to 1.14 gigatons CO2 emissions in 2015. *One Earth* **5**, 1165–1177 (2022).

22. T.-C. Lirn, R.-D. Wong, Determinants of grain shippers' and importers' freight transport choice behaviour. *Prod. Plan. Control* **24**, 575–588 (2013).

23. F. Medda, L. Trujillo, Short-sea shipping: An analysis of its determinants. *Marit. Policy Manag.* **37**, 285–303 (2010).

24. J. Fry, K. Kanemoto, A. Fraser, K. Nansai, Global freight transport emissions responsibility. *Environ. Sci. Technol.* **58**, 19231–19242 (2024).

25. UNCTAD, Review of Maritime Transport 2024. https://unctad.org/system/files/official-document/rmt2024_en.pdf (2024).

26. S. N. Sirimanne, J. Hoffman, W. Juan, R. Asariotis, M. Assaf, G. Ayala, H. Benamara, D. Chantrel, J. Hoffmann, A. Premti, Review of maritime transport 2019. in *United Nations conference on trade and development, Geneva*, Switzerland. (2019), vol. 9.

27. S. K. Afesorgbor, The impact of economic sanctions on international trade: How do threatened sanctions compare with imposed sanctions? *Eur. J. Political Econ.* **56**, 11–26 (2019).

28. L. Bertassello, P. Winters, M. F. Müller, Access to global wheat reserves determines country-level vulnerability to conflict-induced Ukrainian wheat supply disruption. *Nat. Food* **4**, 673–676 (2023).

29. P. D. Fajgelbaum, A. K. Khandelwal, The economic impacts of the US–China trade war. *Annu. Rev. Econ.* **14**, 205–228 (2022).

30. M. Jakob, Climate policy and international trade–A critical appraisal of the literature. *Energ. Policy* **156**, 112399 (2021).

31. A. Bottasso, M. Conti, P. C. de Sa Porto, C. Ferrari, A. Tei, Port infrastructures and trade: Empirical evidence from Brazil. *Transp. Res. A: Policy Pract.* **107**, 126–139 (2018).

32. UNCTAD, *Review of maritime transport 2024: Navigating maritime chokepoints* (United Nations, 2024).

33. T. Beaufils, H. Ward, M. Jakob, L. Wenz, Assessing different European Carbon Border Adjustment Mechanism implementations and their impact on trade partners. *Commun. Earth Environ.* **4**, 131 (2023).

34. M. P. Wingender, F. Misch, *Revisiting carbon leakage* (International Monetary Fund, 2021).

35. B. Graver, D. Rutherford, S. Zheng, CO_2 Commercial Aviation 2013,2018,2019. https://theicct.org/sites/default/files/publications/CO2-commercial-aviation-oct2020.pdf (2020).

36. S. Hanaoka, T. Matsuda, W. Saito, T. Kawasaki, T. Hiraide, Identifying factors for selecting land over maritime in inter-regional cross-border transport. *Sustainability* **13**, 1471 (2021).

37. S. Greene, C. Façanha, Carbon offsets for freight transport decarbonization. *Nat. Sustain.* **2**, 994–996 (2019).

38. OECD, CO2 Emissions from Fuel Combustion 2018. https://www.oecd-ilibrary.org/energy/co2-emissions-from-fuel-combustion-2018_co2_fuel-2018-en (2019).

39. Wei, H., Shang, H. M. & Chen, Y. S. Simulating internal combustion engine port-flow using algebraic multi-grid method in a unstructured flow solver. Proceedings of the 1999 3rd ASME/JSME Joint Fluids Engineering Conference, FEDSM'99, San Francisco, California, USA, 18–23 July 1999 (CD-ROM) (1999).

40. W. H. Lam, P. L. Ng, W. Seabrooke, E. C. Hui, Forecasts and reliability analysis of port cargo throughput in Hong Kong. *J. Urban Plann. Dev.* **130**, 133–144 (2004).

41. F.-M. Tsai, L. J. Huang, Using artificial neural networks to predict container flows between the major ports of Asia. *Int. J. Prod. Res.* **55**, 5001–5010 (2017).

42. S.-C. Chen, S.-Y. Kuo, K.-W. Chang, Y.-T. Wang, Improving the forecasting accuracy of air passenger and air cargo demand: The application of back-propagation neural networks. *Transport. Plan. Techn.* **35**, 373–392 (2012).

43. M. F. Loaiza, R. P. Solano, R. Simancas, V. H. Ojito, in *2017 International Conference on Advanced Materials Science and Civil Engineering (AMSCE 2017)*. (Atlantis Press, 2017), pp. 132–137.

44. I. C. Bilegan, T. G. Crainic, M. Gendreau, Forecasting freight demand at intermodal terminals using neural networks–an integrated framework. *Eur. J. Oper. Res* **13**, 22–36 (2008).

45. H. Wu, G. Liu, in *International Conference on Advances in Energy, Environment and Chemical Engineering*. (Atlantis Press, 2015), pp. 449–454.

46. Z. Abdirassilov, A. Sładkowski, Application of artificial neural networks for shortterm prediction of container train flows in direction of China–Europe via Kazakhstan. *Transp. Probl.* **13**, 103–113 (2018).

47. G. Baxter, P. Srisaeng, The use of an artificial neural network to predict Australia's export air cargo demand. *Int. J. Traffic Transp. Eng.* **8**, 15–30 (2018).

48.. Gökkuş, M. S. Yıldırım, M. M. Aydin, Estimation of container traffic at seaports by using several soft computing methods: A case of Turkish Seaports. *Discrete Dyn. Nat. Soc.* **2017**, 2984853 (2017).

49. V. Gosasang, W. Chandraprakaikul, S. Kiattisin, A comparison of traditional and neural networks forecasting techniques for container throughput at Bangkok port. *Asian J. Shipp. Logist.* **27**, 463–482 (2011).

50. M. K. Fung, Forecasting Hong Kong's container throughput: An error-correction model. *J. Forecasting* **21**, 69–80 (2002).

51. Z. Liu, L. Ji, Y. Ye, Z. Geng, Combined forecast method of port container throughput based on RBF neural network. *J. Tongji Univ.* **35**, 739 (2007).

52. D. Wu, X. Pan, in *2010 International Conference on Intelligent Computation Technology and Automation*. (IEEE, 2010), vol. 1, pp. 1035–1038.

53. J. Heaton. Introduction to Neural Networks with Java, Heaton Research (2008).

54. X. Wang, W. Yi, Z. Lv, F. Deng, S. Zheng, H. Xu, J. Zhao, H. Liu, K. He, Ship emissions around China under gradually promoted control policies from 2016 to 2019. *Atmos. Chem. Phys.* **21**, 13835–13853 (2021).

55. UNCTAD. Review of Maritime Transport 2022: Navigating Stormy Waters (2022).

56. B. Zheng, G. Geng, P. Ciais, S. J. Davis, R. V. Martin, J. Meng, N. Wu, F. Chevallier, G. Broquet, F. Boersma, R. van der A, J. Lin, D. Guan, Y. Lei, K. He, Q. Zhang, Satellite-based estimates of decline and rebound in China's CO_2 emissions during COVID-19 pandemic. *Sci. Adv.* **6**, eabd4998 (2020).

57. J. Verschuur, E. E. Koks, J. W. Hall, Ports' criticality in international trade and global supply-chains. *Nat. Commun.* **13**, 4351 (2022).
